# Social Media Intervention Based on the Information-Motivation-Behavioral Skills Model Promotes HIV Testing and Reduces High-Risk Behaviors Among Men Who Have Sex With Men in Resource-Limited Settings in China: Randomized Controlled Trial

**DOI:** 10.2196/84279

**Published:** 2026-04-07

**Authors:** Huanzhuo Mai, Jianyuan Liu, Liping Cheng, Hao Liang, Ping Cui, Qin Yao, Qin Cao, Heng Zhang, Wenping Liao, Shiran He, Xing Yang, Ping Cen, Jiegang Huang

**Affiliations:** 1 School of Public Health, Guangxi Medical University, 22, Shuangyong Road, Nanning, Guangxi, 530021, China, 86 13878833650; 2 Guangxi Key Laboratory of AIDS Prevention and Treatment, Guangxi Medical University, Nanning, Guangxi, China; 3 Key Laboratory of Prevention and Control of Highly Prevalent Diseases (Guangxi Medical University), Education Department of Guangxi Zhuang Autonomous Region, Nanning, Guangxi, China; 4 Life Sciences Institute, Guangxi Medical University, Nanning, Guangxi, China; 5 Nursing College, Guangxi Medical University, Nanning, Guangxi, China; 6 Health Management Research Institute, People's Hospital of Guangxi Zhuang Autonomous Region and Guangxi Academy of Medical Sciences, Nanning, Guangxi, China; 7 Infectious Disease Laboratory, Guangxi AlDS Clinical Treatment Centre (Nanning), The Fourth People's Hospital of Nanning, Nanning, Guangxi, China

**Keywords:** men who have sex with men, social media intervention, information-motivation-behavioral skills model, HIV testing, high-risk behaviors

## Abstract

**Background:**

Social media intervention may enhance HIV prevention among men who have sex with men, but the effect of this intervention in resource-limited settings remains unclear.

**Objective:**

This randomized controlled trial evaluated whether a social media intervention grounded in the information-motivation-behavioral skills (IMB) model could be beneficial for HIV prevention among men who have sex with men in resource-limited settings.

**Methods:**

Participants were recruited in Nanning, China, between April 2023 and April 2024. Eligible participants were randomly assigned to either the social media intervention group or the routine HIV prevention services control group. Participants in the intervention group received a 3-month social media intervention, which included completing video-based tasks. Baseline surveys were conducted, followed by follow-up surveys every 3 months, for a total of 2 follow-ups. Outcomes included HIV testing uptake, high-risk behavior, AIDS-related knowledge, safe sex self-efficacy, and attitude.

**Results:**

A total of 180 eligible men who have sex with men were enrolled (90 per group). Follow-up rates were 97.8% (88/90) and 95.5% (86/90) for the intervention and control groups, respectively. At the follow-ups, the intervention group demonstrated significantly higher uptake of HIV testing, a lower proportion of participants reporting high-risk sexual behaviors, and higher condom use self-efficacy compared to the control group (all *P*<.05). After controlling for sociodemographic variables, generalized estimating equations analysis revealed that the intervention group had significantly higher odds of HIV testing (risk ratio [RR] 1.739, 95% CI 1.110‐2.730), HIV self-testing (RR 2.306, 95% CI 1.593‐3.340), and consistent condom use (RR 2.457, 95% CI 1.636‐3.690) than the control group. Cochran-Armitage trend tests within the intervention group revealed that with increasing intervention duration, both HIV testing and HIV self-testing significantly increased, while high-risk sexual behaviors significantly decreased (all *P*<.05).

**Conclusions:**

The social media intervention guided by the IMB model demonstrated a positive effect on expanding HIV testing coverage, reducing high-risk behavior, enhancing AIDS-related knowledge, and improving safer sex self-efficacy among men who have sex with men in resource-limited settings. These findings provide valuable guidance for future HIV prevention and control efforts targeting this population.

## Introduction

Men who have sex with men represent a high-risk group for HIV infection. As of 2024, men who have sex with men account for 7.6% of global HIV infections among individuals aged 15‐49 years [1]. In China, the HIV infection rate among men who have sex with men is approximately 5.7%, with rates as high as 10.7% in the southwestern region, and infection rates continue to rise [2,3]. A significant proportion of men who have sex with men remain unaware of their HIV status. By 2020, only 62.2% of men who have sex with men in China had ever undergone HIV testing [4,5], which hinders progress toward achieving the first “95” target of “95-95-95” set by the Joint United Nations Programme on HIV/AIDS (UNAIDS)—aiming for 95% of people living with HIV to know their status [6]. Men who have sex with men often engage in behaviors associated with high HIV risk, including unprotected sex, having multiple sexual partners, and recreational drug use [7]. For example, in one study conducted in China, about 37% of men who have sex with men reported engaging in unprotected anal intercourse in the past 6 months [8]. These behaviors increase the likelihood of sexual transmission and exacerbate the epidemic. Reducing such high-risk sexual behaviors can effectively lower the risk of HIV transmission among the men who have sex with men population [9]. Furthermore, they often face social discrimination and stigma [10], creating significant barriers and difficulties to seeking health services and information. These structural and social factors facilitate the spread of HIV within the population. Therefore, implementing effective strategies to improve HIV testing uptake and reduce high-risk behaviors among men who have sex with men is an urgent priority for current HIV prevention and control efforts.

In recent years, social media–based interventions have garnered prominence and have been increasingly adopted for HIV prevention among men who have sex with men [11-13]. Social media refers to various internet-based communication channels, such as social networking sites, blogs, online social media networks, and other forms of communication technology [14]—essentially, digital technology-based platforms using networks for information dissemination. With the rapid advancement of the internet and social media, mobile devices have become ubiquitous personal items. This gives social media interventions the advantage of offering fragmented learning opportunities, addressing limitations of traditional offline interventions [15]. To date, existing studies of social media–based HIV prevention interventions for men who have sex with men in China have largely focused on economically advanced metropolitan regions, including Beijing [16], Anhui [17], and Guangdong [18], which are characterized by relatively high GDP (gross domestic product), well-developed infrastructure, and strong regional influence. In contrast, there is a scarcity of research exploring the effectiveness of such interventions in resource-limited settings within China. Although Nanning serves as the capital of Guangxi, it remains relatively underresourced compared with China’s more developed cities, facing structural and resource constraints in economic development, infrastructure, and research capacity amid ongoing rapid urbanization [19,20]. Therefore, investigating the impact of social media interventions on improving HIV testing uptake and reducing high-risk behaviors among men who have sex with men in these underserved areas is crucial for enhancing local HIV prevention and control efforts.

To maximize the effectiveness of social media interventions, integrating them with established behavioral intervention theories is essential. Compared to other primary behavioral intervention theories [21,22], the information-motivation-behavioral skills (IMB) model, proposed by Fisher, comprehensively considers the influence of psychosocial factors on behavior. It categorizes factors influencing behavior change into 3 components: information, motivation, and behavioral skills. The model posits and validates that possessing disease-prevention information and motivation to engage in prevention can trigger preventive behaviors through behavioral skills to initiate and sustain action [23]. Currently, interventions based on the IMB model are widely applied in HIV behavioral interventions [24,25]. Furthermore, with the rapid development of new media technologies, integrating the IMB model into social media interventions has emerged as a growing trend in public health. Therefore, exploring the feasibility of combining social media interventions with the IMB model holds significant importance for developing more effective HIV prevention and control strategies.

Previous studies in China have demonstrated that social media–based interventions can improve HIV testing uptake among men who have sex with men, but evaluations have primarily focused on economically developed metropolitan areas [16-18]. Although this approach has been shown to increase HIV testing, evidence regarding its effectiveness in reducing high-risk behaviors remains limited. Moreover, in prior studies, intervention effects have mostly been limited to increasing HIV testing willingness or behavior, while effects on promoting broader key behavioral changes such as condom use and pre-exposure prophylaxis (PrEP) adoption remain unclear; most studies only conducted online interventions and data collection, making it difficult to ensure the quality of online information; interventions often lacked grounding in established behavioral change theories; furthermore, outcomes were often measured only at the end of the intervention period without follow-up assessments to determine whether the effects were sustained over time [16-18]. Guangxi, a resource-limited province in southern China, has one of the highest HIV burdens nationwide, particularly among men who have sex with men [26,27]. To address these gaps, we conducted a 2-arm, parallel-group randomized controlled trial (RCT) in Guangxi, with additional emphasis on outcomes related to promoting broader key behavioral changes such as condom use and PrEP adoption, incorporated offline data collection methods to ensure quality, and the application of the IMB model to guide the intervention process, in order to evaluate whether a social media–based intervention could both increase HIV testing and reduce high-risk behaviors. Furthermore, a continued follow-up assessment was incorporated after the intervention to evaluate the durability of the intervention effects over time. This study aimed to provide context-specific evidence to evaluate a social media–based intervention’s efficacy and sustainability in increasing HIV testing and reducing high-risk behaviors among men who have sex with men, thereby guiding more targeted and scalable HIV prevention strategies for men who have sex with men, supporting optimal allocation of limited health resources in comparable settings, and contributing to the achievement of the 95-95-95 targets.

## Methods

### Trial Design and Participants

This study used a 2-armed, parallel-group RCT design. Recruitment of participants among men who have sex with men was conducted using a combination of convenience sampling and peer-driven sampling methods through Guangxi Caihong, a gay-led community-based organization (CBO) based in Nanning, China. On-site peer educators posted online recruitment notices and registration questionnaires on social platforms such as WeChat Moments, WeChat Official Accounts, WeChat/QQ groups, and Blued. Individuals who completed online registration underwent an initial screening by staff, and the results were notified via SMS text message. Those who passed the initial screening were required to arrive at the CBO site to proceed with the next steps of the survey. Participants who were selected and completed the on-site survey received a cash compensation of RMB 20 (approximately US $3), regardless of whether they were eventually enrolled in the study. Additionally, men who have sex with men who had registered, participated, or were subsequently enrolled were encouraged to refer friends or partners who met the inclusion criteria to join the study.

**Figure 1.**
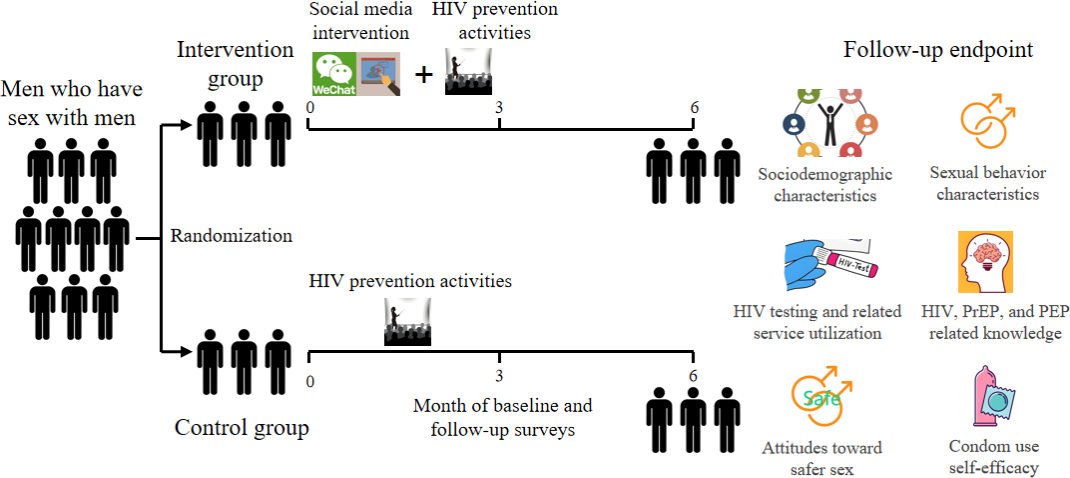
Trial design diagram. The study used a 2-armed, parallel-group randomized controlled trial design. Participants were recruited from among men who have sex with men through the Guangxi Caihong organization in Nanning, China. After recruitment and baseline survey, a 3-month intervention period commenced, with follow-up assessments conducted at the 3rd and 6th months (focusing on the 6 assessment categories shown under “follow-up endpoint” in the figure). Both the intervention and control groups participated in HIV prevention activities over 3 months, while the intervention group additionally received a social media–based intervention. PEP: post-exposure prophylaxis; PrEP: pre-exposure prophylaxis.

The entire social media intervention procedure was conducted online via WeChat, the predominant social media platform in China [28]. Other research procedures, including informed consent, baseline assessment, and follow-up, were carried out offline. The 2-armed study design was (1) social media intervention group and (2) routine HIV prevention (control) services group. All participants completed assessments at 3 time points: baseline (preintervention), 3 months postbaseline (postintervention), and 6 months postbaseline (3 months after postintervention; Figure 1).

Participants were eligible if they met the following criteria: (1) born biologically male, (2) aged 18‐45 years, (3) current Nanning residents planning to reside here for most of the next year, (4) self-reported male-to-male sex in the past 6 months, (5) proficient in using WeChat and willing to connect with the study account, (6) tested HIV-negative within the past 3 months (with test results provided by participants or verified on-site by CBO), and (7) demonstrated sufficient comprehension to complete study procedures. Individuals who had significant psychological, psychiatric, and intellectual disabilities preventing survey participation, or who were currently participating in other related intervention studies, were excluded.

We followed the standard guidelines for reporting parallel group RCTs of CONSORT (Consolidated Standards of Reporting Trials) 2010 [29]. Further details regarding study design and intervention development are provided in Multimedia Appendix 1.

### Sample Size

The sample size was calculated for this on-site RCT based on previous similar studies. The calculation formula is as follows:


$$N=\frac{Z_{\alpha }\sqrt{2\bar{P}(1-\bar{P})}+Z_{\beta }\sqrt{P_{1}(1-P_{1})+P_{2}(1-P_{2})}}{(P_{1}-P_{2}{)}^{2}}$$

The study has 2 primary outcomes: HIV testing rate and the incidence of high-risk sexual behaviors. Previous studies have shown that digital media interventions significantly increase HIV testing rates or HIV self-testing rates (by 48.7%‐63%) [16,17]. Meanwhile, prior research has reported a relatively small expected difference in the incidence of high-risk sexual behaviors (17%‐35%) [9,30]. According to the RCT sample size formula, calculating based on the incidence of high-risk sexual behaviors yields a larger required sample size. To ensure sufficient study power and reflect the most conservative estimate, this study’s sample size was determined based on the expected difference in the incidence of high-risk sexual behaviors between the two men who have sex with men groups.

Previous studies had reported that 25%‐42% of men who have sex with men engaged in high-risk sexual behaviors in the past 6 months [8,30]. Based on this evidence, we assumed a baseline prevalence (*ρ*) of 35%. Prior interventions had demonstrated reductions of 17%‐35% in such behaviors [9,31]. To ensure a conservative estimate, we assumed a 15% reduction, setting the expected prevalence in the intervention group (*P*_1_) at 20% and in the control group (*P*_2_) at 35%. With a significance level (α) of .05 (*Z*_α_=1.96) and power (1 – β) of 80% (*Z*_β_=0.84), the required sample size per group was calculated as n=78. Accounting for an estimated 10% loss to follow-up, we planned to recruit 90 participants per group, yielding a total of 180 men who have sex with men.

### Randomization and Blinding

To ensure the privacy of study participants, each was assigned a unique identification number for all subsequent follow-ups. For randomization, the allocation sequence was generated by an independent researcher, who was not involved in participant recruitment or any subsequent intervention procedures, using the “randomizr” package in R software (R Foundation for Statistical Computing). Participants were then randomly allocated in a 1:1 ratio to either the intervention or control group based on their assigned identification numbers. Participants were blinded to their group assignment and instructed to maintain confidentiality regarding their intervention status. Throughout the recruitment, baseline assessment, and all follow-up stages, the research staff responsible for these tasks remained unaware of the group allocations, thereby ensuring allocation concealment. While research staff administering the interventions were necessarily unblinded, the statisticians conducting data analysis remained blinded to group allocations throughout the study to ensure objective outcome assessment.

### Intervention Exposure

All participants added the study team’s WeChat work account for information notifications. The control arm received routine HIV prevention services provided by Guangxi Caihong. These services, conducted offline, included HIV/AIDS education and awareness campaigns, regular HIV testing services, and structured peer education sessions, held at least once a month.

Participants in the intervention group received the same services as the control group, with the addition of a social media–based intervention. This intervention was delivered via “Rainbow Home,” a WeChat mini-program developed by the research team. The research team assisted participants in the intervention group with guided registration, provided a detailed explanation of the mini-program’s features, and conducted weekly check-in tasks (Multimedia Appendix 2). To minimize intervention contamination between groups, researchers provided both verbal and written instructions to all participants in the intervention group before the intervention began, requesting that they refrain from sharing the mini-program or any task-related content with others until the end of the study period. This request did not impose any coercion or restriction on the participants. Furthermore, the WeChat mini-program used in this study was configured with restricted access and anonymous log-in. Access privileges were granted exclusively to the intervention group participants by the researchers via dynamic invitation codes, ensuring that unauthorized individuals could not use the mini-program. Participants in the intervention group were unable to view any information about other members within their group. Additionally, service schedules of the intervention group differed from the control group, while all other elements—including activity content and study personnel—remained consistent across both groups.

The intervention based on the IMB model was structured around 4 core components: information, motivation, behavioral skills, and behavior. Content mainly contained comprehensive HIV/AIDS knowledge, real-world case studies highlighting HIV infection risks and prevention narratives, practical guidance on safer sexual practices and correct use of prevention tools, and provision of free HIV self-testing kits. See Table 1 for detailed intervention content.

**Table 1.** The detailed intervention content based on the information-motivation-behavioral skills (IMB) model^a^.

Component	Content
Information	Delivered knowledge via animated videos on HIV/AIDS, sexually transmitted infections, correct HIV testing and self-testing, proper use of PrEP^b^ and PEP^c^, correct condom use, recreational drug side effects, and HIV case studies with treatment.
Motivation	Raised awareness of safer sex and self-protection through sharing HIV infection case studies and provided one-on-one psychological counseling to participants in need.
Behavioral skills	Weekly video-based check-in tasks were sent via mini-program, with staff available for consultation and tailored advice. Free HIV self-test kits with usage guidance were provided, and partner testing was encouraged.
Behavior	Change attitudes toward safer sex, improve self-efficacy in condom use, promote intentions to engage in safer sexual practices, increase awareness of HIV testing, and reduce high-risk sexual behaviors.

a Guided by the IMB model, the intervention content was structured around the 4 core components of the model: information, motivation, behavioral skills, and behavior.

b PrEP: pre-exposure prophylaxis.

c PEP: post-exposure prophylaxis.

The intervention was delivered primarily through video viewing; for example, procedural knowledge (such as PrEP usage guidelines and HIV self-testing procedures) was provided through animated videos. Research staff released 1‐2 video-based tasks per week (a total of 12 tasks) via the mini-program backend. The main content, video duration of each task, and its alignment with the 4 components based on the IMB model are provided in Multimedia Appendix 3. Tasks were pushed every Sunday at 7:30 PM and remained accessible for 2 weeks to allow flexibility in completion. Automated reminder notifications were sent through WeChat, and participants completing all tasks within the 3-month intervention period received financial incentives. In parallel, trained staff provided real-time consultation through WeChat, addressing participant inquiries and encouraging feedback on platform usability. At the 3- and 6-month follow-ups, intervention participants were encouraged to conduct HIV testing on-site, request free HIV self-testing kits by mail or on-site pickup, and invite their sexual partners to undergo testing.

During the follow-up process, for participants suspected of being HIV-positive, the research team members provided them with corresponding psychological support and medical referral services, assisting them in visiting the local Centers for Disease Control or designated hospitals for further confirmatory testing and subsequent follow-up management. Once a participant was confirmed positive, it was considered an endpoint event, and further follow-up was discontinued from the date of diagnosis.

The intervention in this study was conducted in accordance with the research protocol. After the intervention materials were developed, they were reviewed by 3 public health experts and peer educators before being finalized and disseminated. All research staff received standardized training prior to the intervention, and they documented and reported the implementation of interventions in real time. Dedicated staff monitored each participant’s intervention engagement and task completion rate via the system backend, and backend monitoring data were exported and evaluated every 2 weeks.

After the conclusion of this study, participants in the control group will be granted the same access to the WeChat mini-program and all intervention content as the intervention group. However, the program still requires further optimization and is not currently intended for public release.

### Outcomes, Data Collection, and Management

The primary outcome was the uptake of HIV testing and HIV self-testing (verified through testing at the CBO and photo-submitted self-test results to the staff at the 3-month and 6-month follow-ups after enrollment, respectively), along with self-reported high-risk behaviors among men who have sex with men in the past 3 months (defined as engaging in anal intercourse with a male partner without consistent condom use throughout the act or experiencing condom breakage during intercourse). Secondary outcomes included the uptake of syphilis testing (verified through testing at the CBO), as well as knowledge of AIDS, PrEP, and post-exposure prophylaxis (PEP), self-efficacy for condom use, and safe sex attitudes. Validated scales were used to measure knowledge, self-efficacy, and safe sex attitudes. Definitions of outcomes and methods of outcome assessment were detailed in Multimedia Appendix 4.

All self-reported outcomes were evaluated via follow-up surveys administered at enrollment and at 3 and 6 months postenrollment, conducted offline at the CBO site through one-on-one interviews by research staff for data collection.

All original questionnaires and backend-exported data were independently double-entered by two researchers after quality control checks. Personal information was removed, and the data were stored on an encrypted hard drive accessible only to the principal investigator of the research team. The data used during the analysis phase were anonymized. Both the original paper questionnaires and backup electronic questionnaire files are stored in the research team’s encrypted hard drive.

### Statistical Analysis

Descriptive statistics were used to summarize the baseline characteristics. Continuous variables are presented as mean (SD) and were compared using *t* tests or ANOVA. Categorical variables are presented as frequency (percentage) and were compared using chi-square tests or Fisher exact test. The Cochran-Armitage trend test was used for repeated categorical measures within the intervention group. To investigate the effect of the intervention, generalized estimating equations (GEE) were used to analyze correlations for repeated measures data (eg, HIV testing and condom use) across baseline and follow-up time points and between intervention and control groups. Bonferroni correction was applied for post hoc pairwise comparisons. All tests were 2-tailed, with a significance level defined as α=.05; *P* values <.05 were considered statistically significant. All data analyses were completed using R Studio (version 4.2.1; Posit Software) or SPSS (version 26.0; IBM Inc).

### Ethical Considerations

This RCT was retrospectively registered at the Chinese Clinical Trial Registry (ChiCTR2500095176). Ethical approval was obtained from the Medical Ethics Committee of Nanning Center for Disease Control and Prevention (2023014) and the Medical Ethics Committee of Guangxi Medical University (2024-KY0148).

Following ethics committee approval, the study was submitted to the Chinese Clinical Trial Registry. However, due to the platform’s review process and timeline, participant recruitment began under the ethics-approved protocol before the official trial registration number was issued, resulting in a retrospective registration. Throughout the study, all procedures adhered strictly to the original ethics-approved protocol, with no changes to the study content or primary outcomes before or after registration.

Participation in this study was voluntary, and written informed consent was provided by all participants. All analytical data were processed anonymously to ensure participant confidentiality. All research staff committed to not disclosing any participant information. At baseline and each follow-up, participants received a cash compensation of RMB 20 (approximately US $3) upon completing the survey. Additionally, those who completed all tasks during the intervention period were provided an extra cash compensation of RMB 20 (approximately US $3).

## Results

### Participant Flow and Intervention Adherence

**Figure 2.**
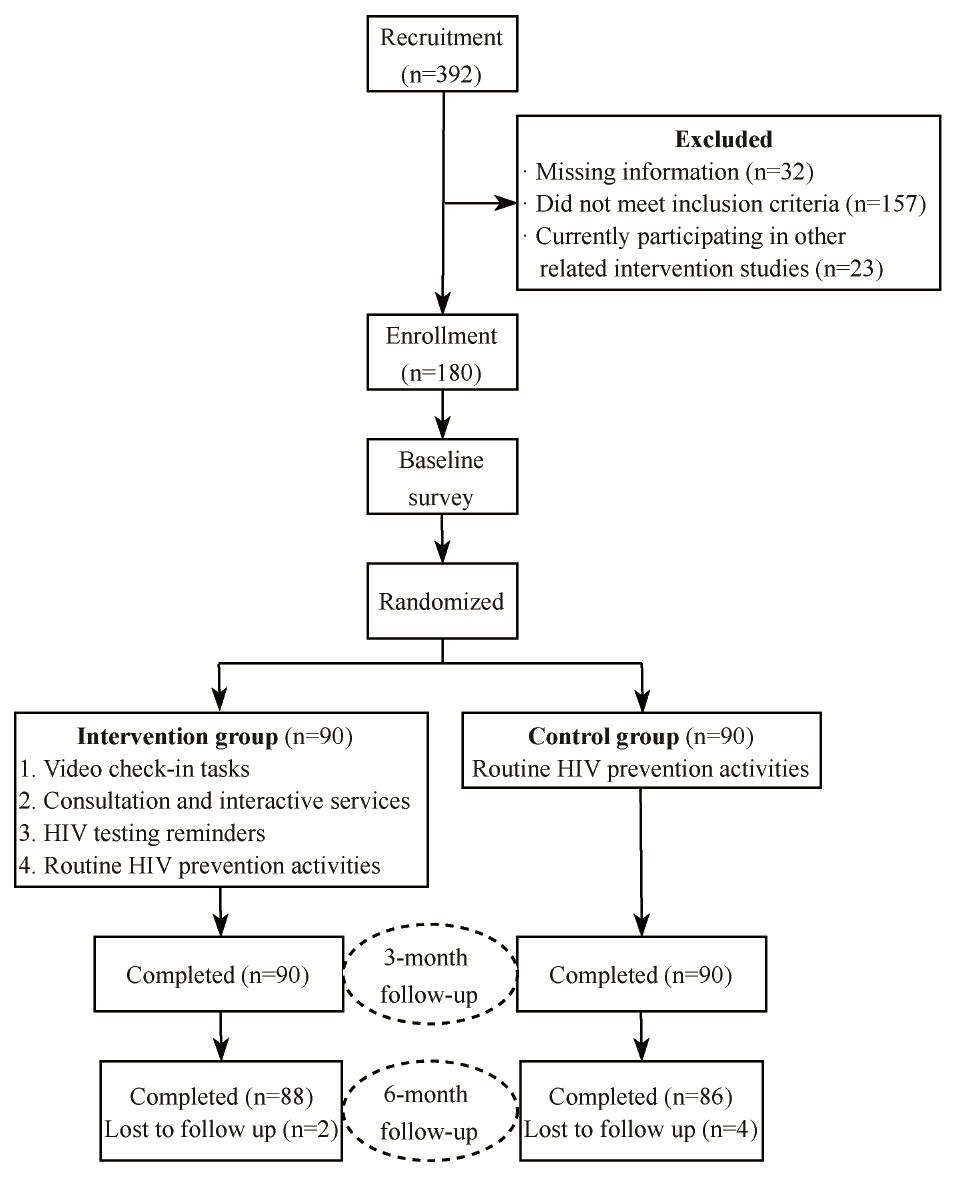
CONSORT (Consolidated Standards of Reporting Trials) flow diagram. Inclusion criteria: (1) born biologically male, (2) aged 18-45 years, (3) current Nanning residents planning to reside here for most of the next year, (4) self-reported male-to-male sex in the past 6 months, (5) proficient in using WeChat and willing to connect with the study account, (6) tested HIV-negative within the past 3 months, and (7) demonstrated sufficient comprehension to complete study procedures.

Recruitment took place between April 2023 and August 2023, during which 392 men who have sex with men were initially screened. After applying the inclusion and exclusion criteria, 180 eligible participants were enrolled and randomly allocated to either the social media intervention group (n=90) or the routine HIV prevention control group (n=90). The formal intervention began in September 2023, and all follow-ups were completed by April 2024. All participants were retained at the 3-month follow-up assessment. At the 6-month follow-up, loss to follow-up occurred in 2 participants (2/90, 2.2%) from the intervention group and 4 participants (4/90, 4.5%) from the control group, yielding follow-up rates of 97.8% (88/90) and 95.5% (86/90), and an overall follow-up rate of 96.7% (174/180). The participant recruitment, intervention, and follow-up flowchart is detailed in Figure 2. During the 3-month intervention period, 12 video check-in tasks were delivered to the intervention group. The average duration per video task was 112 seconds. Participants completed a total of 861 check-ins, representing a task completion rate of 79.7% (861/1080). Due to the low proportion of missing data, no data imputation was performed in this study. The analysis of this study was based on complete case analysis, including only participants with complete outcome data (ie, those who completed all follow-ups).

### Baseline Characteristics of Participants

The mean age of participants was 28.5 (SD 6.9) years. The majority (143/180, 79.4%) had an educational level of college/university or above. Most participants were employed in government institutions or as office workers (91/180, 50.6%). Regarding sexual behavior characteristics, 68.3% (123/180) identified as homosexual in orientation. More than half (104/180, 57.8%) reported engaging in high-risk sexual behaviors. Of the 180 participants, 106 (58.9%) reported having performed HIV self-testing in the past 3 months. After randomization, there were no statistically significant differences in demographic or behavior characteristics between the two groups, indicating comparability (Table 2).

**Table 2.** Sociodemographic and behavioral characteristics of study participants.

Characteristics	Total (n=180), n (%)	Control group (n=90), n (%)	Intervention group (n=90), n (%)	Chi-square (*df*)	*P* value
Age (year)				0.7 (2)	.70
≤24	51 (28.3)	23 (25.6)	28 (31.1)		
25-34	87 (48.3)	45 (50.0)	42 (46.7)		
≥35	42 (23.3)	22 (24.4)	20 (22.2)		
Household registration				0.4 (2)	.82
Nanning City	84 (46.7)	40 (44.4)	44 (48.9)		
Other cities within the province	80 (44.4)	42 (46.7)	38 (42.2)		
Other cities outside the province	16 (8.9)	8 (8.9)	8 (8.9)		
Ethnicity				2.1 (2)^a^	.34
Han	100 (55.6)	47 (52.2)	53 (58.9)		
Zhuang	75 (41.7)	39 (43.3)	36 (40.0)		
Other	5 (2.8)	4 (4.4)	1 (1.1)		
Education level				2.3 (2)^a^	.34
Junior high school or below	9 (5.0)	6 (6.7)	3 (3.3)		
Vocational school/high school	28 (15.6)	11 (12.2)	17 (18.9)		
College/bachelor's degree or higher	143 (79.4)	73 (81.1)	70 (77.8)		
Occupation				4.1 (4)^a^	.38
Student	35 (19.4)	15 (16.7)	20 (22.2)		
Public institution/staff	91 (50.6)	48 (53.4)	43 (47.8)		
Farmer/worker	11 (6.1)	3 (3.3)	8 (8.9)		
Self-employed	24 (13.3)	12 (13.3)	12 (13.3)		
Unemployed	19 (10.6)	12 (13.3)	7 (7.8)		
Monthly income (CNY)				0.7 (2)	.70
<3000 (US $435)	63 (35.0)	32 (35.6)	31 (34.4)		
3000-5000 (US $435-$725)	67 (37.2)	31 (34.4)	36 (40.0)		
>5000 (US $725)	50 (27.8)	27 (30.0)	23 (25.6)		
Marital status				1.2 (2)	.55
Unmarried	160 (88.8)	78 (86.7)	82 (91.1)		
Married	10 (5.6)	7 (7.8)	3 (3.3)		
Divorced	10 (5.6)	5 (5.5)	5 (5.6)		
Living arrangement				0.6 (2)	.75
Living alone	95 (52.8)	47 (52.2)	48 (53.3)		
Living with family/partner	42 (23.3)	23 (25.6)	19 (21.1)		
Dormitory/shared rental	43 (23.9)	20 (22.2)	23 (25.6)		
Sexual orientation				1.1 (2)^a^	.59
Homosexual	123 (68.3)	60 (66.7)	63 (70.0)		
Bisexual	49 (27.2)	27 (30.0)	22 (24.4)		
Uncertain	8 (4.4)	3 (3.3)	5 (5.6)		
Sexual role				0.1 (2)	.96
Insertive role	48 (26.7)	23 (25.6)	25 (27.8)		
Receptive role	65 (36.1)	33 (36.7)	32 (35.6)		
Versatile	67 (37.2)	34 (37.8)	33 (36.7)		
Age at first anal sex (years)				0.8 (1)	.45
≤18	74 (41.1)	34 (37.8)	40 (44.4)		
>18	106 (58.9)	56 (62.2)	50 (55.6)		
Number of regular partners				0.5 (2)	.85
0	50 (27.8)	27 (30.0)	23 (25.6)		
1-2	120 (66.7)	58 (64.4)	62 (68.9)		
≥3	10 (5.6)	5 (5.6)	5 (5.6)		
Number of casual partners				2.0 (2)	.38
0	77 (42.8)	34 (37.8)	43 (47.8)		
1-2	46 (25.6)	24 (26.7)	22 (24.4)		
≥3	57 (31.7)	32 (35.6)	25 (27.8)		
Awareness of partner's HIV status				2.3 (1)	.13
No	78 (43.3)	44 (48.9)	34 (37.8)		
Yes	102 (56.7)	46 (51.1)	56 (62.2)		
Sexting				1.1 (1)	.39
No	46 (25.6)	26 (28.9)	20 (22.2)		
Yes	134 (74.4)	64 (71.1)	70 (77.8)		
Popper/alkyl nitrite inhalant use				0.3 (1)	.62
No	131 (72.8)	64 (71.1)	67 (74.4)		
Yes	49 (27.2)	26 (28.9)	23 (25.5)		
Consistent condom use				3.3 (1)	.10
Yes	76 (42.2)	32 (35.6)	44 (48.9)		
No	104 (57.8)	58 (64.4)	46 (51.1)		
HIV self-testing				0.4 (1)	.65
No	74 (41.1)	35 (38.9)	39 (43.3)		
Yes	106 (58.9)	55 (61.1)	51 (56.7)		
Syphilis test result				1.6 (1)	.31
Negative	163 (90.6)	84 (93.3)	79 (87.8)		
Positive	17 (9.4)	6 (6.7)	11 (12.2)		
HIV testing				1.5 (1)	.15
No	43 (23.9)	18 (20.0)	25 (27.8)		
Yes	137 (76.1)	72 (80.0)	65 (72.2)		

a Fisher exact test.

### Social Media Interventions Drive Increases Testing and Reductions in High-Risk Behavior Among Men Who Have Sex With Men

At the 3-month follow-up, the HIV self-testing rate was significantly higher in the intervention group than in the control group (*P*<.001), and the proportion of participants reporting high-risk sexual behaviors was significantly lower (*P*<.05). The number of casual partners and the rate of popper/alkyl nitrite inhalant use in the intervention group were significantly lower than in the control group (*P*<.05). The intervention group had a significantly higher rate of awareness of their sexual partners’ HIV status compared to the control group (*P*<.05; Table 3).

**Table 3.** Comparison of sexual behavior characteristics and HIV testing status between the intervention group and the control group among study participants after 6-month follow-up.

Variables and groups	Baseline (intervention: n=90; control: n=90), n (%)	3-month follow-up (intervention: n=90; control: n=90), n (%)	6-month follow-up (intervention: n=88; control: n=86), n (%)
HIV testing			
Intervention	65 (72.2)	74 (82.2)	81 (92.0)^a,b^
Control	72 (80.0)	66 (73.3)	56 (65.1)
HIV self-testing			
Intervention	51 (56.7)	63 (70.0)^a^	63 (71.6)^a,c^
Control	55 (61.1)	31 (34.4)	32 (37.2)
Syphilis test result (positive)			
Intervention	11 (12.2)	3 (3.3)	4 (4.5)
Control	6 (6.7)	13 (14.4)	7 (8.1)
Number of regular partners^d^			
0			
Intervention	23 (25.6)	24 (26.7)	25 (28.4)
Control	27 (30.0)	33 (36.7)	21 (26.7)
1‐2			
Intervention	62 (68.9)	69 (76.7)	60 (68.2)
Control	58 (64.4)	52 (57.8)	61 (76.7)
≥3			
Intervention	5 (5.6)	5 (5.6)	3 (3.4)
Control	5 (5.6)	5 (5.6)	4 (5.5)
Number of casual partners^e^			
0			
Intervention	43 (47.8)	54 (60.0)	38 (43.2)
Control	34 (37.8)	46 (51.1)	47 (60.0)
1‐2			
Intervention	22 (24.4)	31 (34.4)	32 (36.4)
Control	24 (26.7)	30 (33.3)	31 (34.4)
≥3			
Intervention	25 (27.8)	11 (12.2)	13 (14.8)
Control	32 (35.6)	14 (15.5)	8 (12.2)
Awareness of partner’s HIV status^f^			
Intervention	56 (62.2)	69 (76.7)^d^	62 (74.7)^a^
Control	46 (51.1)	50 (62.5)	40 (49.4)
High-risk sexual behavior^f^			
Intervention	46 (51.1)	33 (36.7)^d^	26 (31.3)^g^
Control	58 (64.4)	45 (55.5)	36 (44.4)
Sexting			
Intervention	70 (77.8)	63 (70.0)	56 (63.6)^c^
Control	64 (71.1)	57 (63.3)	58 (67.4)
Popper/alkyl nitrite inhalant use			
Intervention	23 (25.5)	16 (17.8)^d^	12 (13.6)^c,e^
Control	26 (28.9)	31 (34.4)	26 (30.2)

a *P*<.001 compared to the control group.

b *P*<.001 compared to the baseline.

c *P*<.05 compared to the baseline.

d *P*<.05 compared to the control group.

e *P*<.01 compared to the control group.

f There are missing values as some study participants had no sexual partners in the last 3 months and were therefore excluded from the analysis.

g *P*<.01 compared to the baseline.

At the 6-month follow-up, both HIV testing rate and HIV self-testing rate were significantly higher in the intervention group compared to the control group (*P*<.001). The proportions of participants reporting high-risk sexual behaviors and popper/alkyl nitrite inhalant use were lower in the intervention group than in the control group (*P*<.05). Additionally, participants in the intervention group were significantly more likely to be aware of their sexual partners’ HIV status than those in the control group (*P*<.001). Regarding sexually transmitted infections, 16 cases of syphilis were detected at the 3-month follow-up (3 in the intervention group and 13 in the control group), and 11 cases were identified at the 6-month follow-up (4 in the intervention group and 7 in the control group). No participants tested positive for HIV at either follow-up assessment (Table 3).

Cochran-Armitage trend tests within the intervention group revealed significant linear trends over time for all measured variables except number of regular sexual partners, number of casual sexual partners, and awareness of partner HIV status (*P*<.05). These variables included HIV testing, HIV self-testing, high-risk sexual behaviors, sexting behavior, and popper/alkyl nitrite inhalant use. Specifically, the positive effects observed in the intervention group at month 3 (the end of the intervention)—such as the promotion of HIV testing and HIV self-testing, reduction of high-risk sexual behaviors, and reduction of Rush popper use—were maintained and even strengthened at the 6-month follow-up assessment (3 months after the intervention ended; Table 3).

After controlling for sociodemographic variables, repeated measures ANOVA (with time as the within-subject factor and group as the between-subject factor) revealed: The intervention group had significantly higher odds of HIV testing (risk ratio [RR] 1.739, 95% CI 1.110‐2.730), HIV self-testing (RR 2.306, 95% CI 1.593‐3.340), and consistent condom use (RR 2.457, 95% CI 1.636‐3.690) compared to the control group. The intervention group had significantly lower odds of popper/alkyl nitrite inhalant use compared to the control group (RR 0.554, 95% CI 0.365‐0.841). Compared to the control group, the intervention group showed significantly increased odds of HIV self-testing at the 3-month follow-up (RR 5.651, 95% CI 2.321‐13.761); also, significantly increased odds of both HIV testing (RR 10.473, 95% CI 3.276‐33.491) and HIV self-testing (RR 5.651, 95% CI 2.321‐13.761) were observed at the 6-month follow-up (Table 4).

**Table 4.** Analysis of the impact of new media intervention on high-risk sexual behaviors and HIV testing before and after intervention^a^.

Variable	β value	RR^b^ (95% CI)	*P* value^c^
HIV testing (no=0, yes=1)			
Group (intervention vs control)	0.553	1.739 (1.110‐2.730)	*.02*
Time1 (3-month follow-up)	0.208	1.187 (0.709‐1.990)	.51
Time2 (6-month follow-up)	0.172	0.475 (0.732‐2.070)	.43
Group * Time1	0.893	2.443 (0.876‐6.808)	.09
Group * Time2	2.349	10.473 (3.276‐33.491)	*<.001*
HIV self-testing (no=0, yes=1)			
Group (intervention vs control)	0.835	2.306 (1.593‐3.340)	*<.001*
Time1 (3-month follow-up)	−0.225	0.798 (0.512‐1.250)	.32
Time2 (6-month follow-up)	−0.151	0.860 (0.551‐1.340)	.51
Group * Time1	1.732	5.651 (2.321‐13.761)	*<.001*
Group * Time2	1.686	5.395 (2.221‐13.117)	*<.001*
Consistent condom use^d^ (no=0, yes=1)			
Group (intervention vs control)	0.899	2.457 (1.638‐3.687)	*<.001*
Time1 (3-month follow-up)	0.699	2.011 (1.252‐3.233)	*.004*
Time2 (6-month follow-up)	0.873	2.395 (1.475‐3.885)	*<.001*
Group * Time1	−0.222	0.801 (0.310‐2.068)	.65
Group * Time2	0.013	1.013 (0.382‐2.683)	.98
Sexting (no=0, yes=1)			
Group (intervention vs control)	0.242	0.785 (0.530‐1.163)	.23
Time1 (3-month follow-up)	−0.509	0.636 (0.375‐0.964)	*.04*
Time2 (6-month follow-up)	−0.564	0.748 (0.351‐0.923)	*.02*
Group * Time1	−0.125	0.882 (0.340‐2.290)	.80
Group * Time2	−0.560	0.571 (0.216‐1.510)	.26
Popper/alkyl nitrite inhalant use (no=0, yes=1)			
Group (intervention vs control)	−0.591	0.554 (0.365‐0.841)	*.006*
Time1 (3-month follow-up)	−0.031	0.970 (0.595‐1.579)	.90
Time2 (6-month follow-up)	−0.327	1.060 (0.436‐1.193)	.21
Group * Time1	−0.808	0.446 (0.167‐1.190)	.11
Group * Time2	−0.883	0.414 (0.147‐1.160)	.09

a The generalized estimating equations model was adjusted for age, household registration, ethnicity, education level, occupation, and monthly income.

b RR: risk ratio.

c Italic values represent statistically significant at *P*<.05.

d There are missing values as some study participants had no sexual partners within the last 3 months and were therefore excluded from the analysis.

### Social Media Intervention Significantly Increases AIDS-Related Knowledge

Knowledge scores for HIV/AIDS, PrEP, and PEP at baseline, 3 months, and 6 months were presented in Table 5 (mean scores shown). Comparison results indicated that compared to the control group, the intervention group showed significantly higher PrEP knowledge scores at both the 3-month and 6-month follow-ups (both *P*<.05). Furthermore, within the intervention group, knowledge scores for HIV/AIDS, PrEP, and PEP were significantly higher at both follow-up time points compared to baseline (all *P*<.05; Table 5).

**Table 5.** Comparison of awareness levels of relevant health knowledge between the intervention group and the control group before and after intervention.

Variables and groups	Baseline, mean (SD)	3-month follow-up, mean (SD)	6-month follow-up, mean (SD)
AIDS awareness level			
Intervention	13.10 (3.14)	15.10 (2.28)^a,b^	14.50 (2.02)^a,b^
Control	13.10 (3.14)	13.08 (2.32)	13.09 (2.75)
PrEP^c^ awareness level			
Intervention	3.31 (1.56)	5.91 (1.39)^a,b^	6.01 (1.69)^a,b^
Control	3.14 (1.41)	4.87 (2.17)^a^	4.79 (2.38)^a^
PEP^d^ awareness level			
Intervention	5.71 (1.83)	6.71 (1.60)^a,b^	6.68 (1.48)^a,b^
Control	5.58 (1.75)	5.48 (2.17)	5.42 (2.36)

a *P*<.05 compared to the baseline.

b *P*<.05 compared to the control group.

c PrEP: pre-exposure prophylaxis.

d PEP: post-exposure prophylaxis.

### Social Media Intervention Improves Condom Use Self-Efficacy and Modifies Sex Attitudes in Men Who Have Sex With Men

Scores for condom use self-efficacy and safer sex attitudes at baseline, 3 months, and 6 months are presented in Table 6 (mean scores shown). Results demonstrated that compared to the control group, condom use self-efficacy was significantly higher in the intervention group at the 3-month follow-up (*P*<.05). Within the intervention group, condom use self-efficacy scores increased significantly from baseline to both the 3-month and 6-month follow-ups (both *P*<.05), with scores at 6 months being significantly higher than those at 3 months (*P*<.05; Table 6).

**Table 6.** Comparison of safe behavior attitudes and self-efficacy in condom use between the intervention group and the control group before and after intervention.

Variables and groups	Baseline, mean (SD)	3-month follow-up, mean (SD)	6-month follow-up, mean (SD)
Condom use self-efficacy			
Intervention	30.18 (6.27)	32.29 (5.38)^a,b^	33.21 (5.06)^a,c^
Control	29.03 (6.24)	30.82 (4.83)^a^	32.89 (5.83)^a,c^
Safe behavior attitudes			
Intervention	45.95 (8.65)	47.74 (7.82)	47.65 (7.20)
Control	45.39 (8.06)	46.73 (7.57)	46.87 (7.38)

a *P*<.05 compared to the control group.

b *P*<.05 compared to the baseline.

c *P*<.05 compared to the 3-month follow-up.

## Discussion

Concerning the impact of the social media intervention on promoting HIV testing among men who have sex with men, this study found a higher uptake of HIV testing in the intervention group compared to the control group. This finding aligns with the results of some previous similar studies, such as two WeChat-based interventions conducted in Anhui, China, and Guangdong, China, where the HIV testing rates in the intervention groups were significantly higher than those in the control groups [17,18]. Our research further confirms the effectiveness of WeChat-based social media interventions in promoting HIV testing among the men who have sex with men population, indicating a positive effect of the social media–based strategy in increasing HIV testing coverage. Particularly for young, internet-savvy men who have sex with men, social media interventions could more effectively convey HIV prevention knowledge and enhance their testing awareness and capacity [32].

Regarding the effect of the social media intervention on reducing high-risk behaviors among men who have sex with men, our results demonstrated that the intervention group exhibited significantly lower frequencies of high-risk sexual behaviors and popper/alkyl nitrite inhalant use compared to the control group. This indicated that the social media intervention effectively reduces high-risk sexual behaviors among men who have sex with men, aligning with findings from multiple similar studies [9,31,33]. It demonstrates the broad application potential of social media interventions in reducing high-risk behaviors and promoting HIV prevention. However, in China, research exploring the impact of WeChat-based social media interventions on reducing high-risk sexual behaviors remains relatively scarce. A WeChat-based intervention study conducted in Anhui Province, China, showed no significant difference in condom use behavior between the intervention and control groups, which may be related to the measures implemented in the control group [17]. This indicates that further and more in-depth research is still needed regarding the effects of WeChat-based social media interventions on reducing high-risk sexual behaviors in China. Furthermore, within the intervention group, after receiving the social media intervention, significant reductions were observed in the frequency of high-risk sexual behaviors, sexting behavior, and popper/alkyl nitrite inhalant use compared to baseline. GEE model analysis confirmed the positive effect of the intervention on reducing high-risk behaviors.

Furthermore, our study found that the positive effects observed in the intervention group at month 3 (the end of the intervention)—such as the promotion of HIV testing and HIV self-testing, reduction of high-risk sexual behaviors, and reduction of Rush popper use compared to the control group—were maintained and even strengthened at the 6-month follow-up assessment (3 months after the intervention ended). This indicates that the social media intervention in this study had sustained effects in promoting risk behavior change among men who have sex with men, continuing to positively influence their health behaviors even after the intervention concluded. This highlights the potential long-term benefits of social media as a health education platform for men who have sex with men, and the important role of social media as a platform for information dissemination and health education.

Our results showed that AIDS-related knowledge levels significantly increased from baseline to postintervention in both groups, with a more pronounced increase in the intervention group. This suggested that, compared to conventional methods, the social media intervention was more effective in enhancing relevant knowledge levels among men who have sex with men. Due to societal discrimination and stigma, especially in resource-limited settings, traditional sexual education and counseling services are often inaccessible or ineffective for men who have sex with men [34,35]. Emerging internet-based social media interventions, popular in men who have sex with men research, use engaging graphics, animations, and short videos to capture attention, foster interactivity and participation, and create a positive atmosphere for interaction. This strengthened men who have sex with men’s understanding and retention of health information, further promoting the dissemination and depth of HIV knowledge delivery. Moreover, PrEP has been proven effective in reducing HIV risk [3]. As a novel and effective educational tool, social media interventions played a crucial role in improving AIDS knowledge, particularly awareness and willingness to use PrEP and PEP, among men who have sex with men. Therefore, future interventions should continue to leverage internet platforms and the strengths of social media to provide more personalized and acceptable services for men who have sex with men.

Using GEE models, this study also evaluated the intervention’s impact on safer sex attitudes and condom use self-efficacy. For condom use self-efficacy, results showed a significant improvement in the intervention group compared to the control group. Within the intervention group, scores significantly increased at both 3 and 6 months compared to baseline. This indicated the positive role of the new media intervention in promoting condom use self-efficacy among men who have sex with men. Bogale et al [36] found significantly increased condom use intention among rural Ethiopian women following an audio-based HIV intervention. Hirshfield and Hennessy et al [37,38] found that television- and radio-based media interventions can increase the intention to use condoms among African American adolescents. Our study further confirms that media-based interventions can enhance individuals’ confidence and capability to use condoms during sex practices. However, no significant differences were found in safer sex attitudes between the groups. This might indicate that men who have sex with men have relatively fixed attitudes toward sexual behavior, shaped by long-term sociocultural influences and personal experiences, which were difficult to change through short-term interventions. Future research could further extend the intervention and follow-up period to confirm its effects on attitudes toward safe sexual behavior.

Our study demonstrates that, compared to routine HIV prevention services alone, a social media–based intervention via WeChat significantly increased HIV testing rates, reduced high-risk sexual behaviors, and enhanced HIV-related knowledge among men who have sex with men. As a leading social media platform in China, WeChat features Mini Programs that require no additional installation, offering low user accessibility barriers [28,39]. This makes it particularly suitable for use in resource-limited settings such as Guangxi. Our research provides novel insights and a reference for integrating social media interventions with routine HIV prevention services to develop diversified strategies in resource-limited regions.

The IMB model categorizes factors influencing behavior change into 3 components: information, motivation, and behavioral skills. It proposes and validates that possessing disease prevention information and having the motivation to engage in prevention can, through behavioral skills, trigger preventive actions to initiate and sustain behavioral change [23]. This study found that interventions based on the IMB model effectively promoted the adoption of HIV preventive behaviors, following the theoretically hypothesized progressive pathway: First, procedural knowledge (such as PrEP usage guidelines and HIV self-testing procedures) provided through animated videos established a solid informational foundation (eg, individuals learned and accumulated knowledge related to HIV testing), while case studies and psychological counseling simultaneously enhanced personal risk perception and awareness of social support, collectively addressing the issues of “knowledge” and “motivation” (eg, individuals recognized the harms of not undergoing HIV testing through case studies, developed a sense of urgency, and strengthened their motivation and support for HIV testing through psychological counseling). Subsequently, skill-building components—such as weekly video check-ins, personalized consultations, and the distribution of free self-testing kits—facilitated the practical mastery of operational skills and improved self-efficacy by creating a low-risk practice environment (eg, individuals learned and mastered the skills for HIV testing). These efforts successfully translated the accumulated information and motivation into actionable behavioral capabilities. Ultimately, this multicomponent synergistic effect drove significant behavioral improvements (eg, individuals ultimately decided to undergo HIV testing). In this study, this was specifically evidenced by increased HIV testing rates, enhanced self-efficacy in condom use, and reduced frequency of high-risk sexual behaviors. Thus, the study validates the utility of the IMB model in constructing effective social media interventions for men who have sex with men populations.

This study holds significant research and policy implications. First, by adopting an RCT design and integrating social media interventions with an established behavior change theory—the IMB model—we were able to more comprehensively account for factors influencing individual behavior change. Second, conducting the research in Guangxi, China—a relatively resource-limited region with a high HIV burden—expands the demographic and geographical diversity of studies on social media–based HIV prevention interventions among men who have sex with men. Third, by using multiple outcome measures to evaluate the intervention’s effectiveness from various dimensions, we demonstrated that our approach effectively promoted HIV testing and reduced high-risk behaviors among men who have sex with men. Fourth, a 3-month postintervention follow-up was conducted, which confirmed the sustained effects of the social media intervention in promoting HIV testing and reducing high-risk behaviors. These findings provide new insights and methodologies for HIV prevention behavioral interventions.

Several limitations should be acknowledged. First, the participants were recruited from one site in China, which may limit the generalizability of the findings due to potential homogeneity within the recruited sample. Second, the survey relied on staff-administered one-on-one interviews, which might introduce discrepancies, especially for sensitive information like sexual behaviors. During follow-up, participants might have been influenced by social desirability bias, potentially underreporting high-risk behaviors and leading to report bias. Social desirability bias can distort the authenticity of data, weaken or even reverse the true relationships between variables. Third, the 6-month duration of this study limited the assessment of long-term effects postintervention. Fourth, although we aimed to reduce the risk of contamination by instructing participants to maintain confidentiality and differentiating service schedules, the use of respondent-driven sampling means that participants in the intervention and control groups may have social connections. Therefore, the possibility of intervention information spreading (contamination) cannot be entirely ruled out theoretically. Fifth, due to the nature of the intervention, complete blinding of participants was challenging, which may have allowed them to deduce their group assignment, potentially introducing expectation bias and reporting bias. Future studies should adopt a combination of strategies—such as implementing “anonymous” designs, using specialized measurement methods like the “randomized response technique,” and incorporating social desirability scales for statistical control during data analysis—to systematically identify and mitigate the impact of social desirability bias, while also expanding recruitment to more diverse geographic regions to verify the effectiveness of social media–based strategies in resource-limited settings, extending the postintervention period of follow-up to examine their sustained impact on HIV prevention behaviors, considering designs such as cluster randomization or randomization based on social network communities to more effectively avoid contamination risks, and implementing a structured active control design to maintain participant blinding.

In conclusion, social media intervention based on the IMB model represented a promising strategy for HIV prevention in resource-limited settings, aimed at men who have sex with men. It significantly contributed to expanding HIV testing coverage, reducing high-risk behavior, enhancing AIDS-related knowledge, and improving safer sex self-efficacy within this high-risk population. Moreover, the positive effects became more pronounced with increasing the intervention and follow-up period. It demonstrated considerable potential and promising application prospects in the broader fight against HIV/AIDS. To achieve the 95-95-95 targets, adapting the IMB model–based social media intervention in low-resource settings and other key populations could prove highly beneficial.

## Supplementary material

10.2196/84279 Multimedia Appendix 1 Intervention development and pilot study.

10.2196/84279 Multimedia Appendix 2 Operational process of WeChat mini-program.

10.2196/84279 Multimedia Appendix 3 Video tasks of the intervention plan based on the information-motivation-behavioral skills model.

10.2196/84279 Multimedia Appendix 4 Definitions of outcomes and methods of outcome assessment.

10.2196/84279 Checklist 1 CONSORT 2010 checklist.

## Abbreviations

CBO: community-based organization
CONSORT: Consolidated Standards of Reporting Trials
GDP: gross domestic product
GEE: generalized estimating equation
IMB: information-motivation-behavioral skills
PEP: post-exposure prophylaxis
PrEP: pre-exposure prophylaxis
RCT: randomized controlled trial
UNAIDS: United Nations Programme on HIV/AIDS
