# Supplementary material for: Social Media Intervention Based on the Information-Motivation-Behavioral Skills Model Promotes HIV Testing and Reduces High-Risk Behaviors Among Men Who Have Sex With Men in Resource-Limited Settings in China: Randomized Controlled Trial
Source: J Med Internet Res. 2026 Apr 7;28:e84279. doi: 10.2196/84279 (PMC13055935; doi:10.2196/84279)
Supplement: Multimedia Appendix 1 [file jmir-v28-e84279-s001.pdf]

Intervention development and pilot study

1. Study Background

In China, social media-based interventions have been proven effective in increasing the HIV testing rate among men who have sex with men (MSM). However, existing research has primarily focused on economically developed cities, and evidence regarding their effectiveness in resource-limited areas is insufficient. Furthermore, the impact of social media interventions on reducing high-risk sexual behaviors remains unclear. This study aims to conduct a randomized controlled trial in Nanning, Guangxi (a resource-limited area), to explore the effects of a social media intervention on HIV testing rates and high-risk sexual behaviors among MSM.

The intervention content was designed based on the Information-Motivation-Behavioral Skills Model (IMB Model). This model integrates three core elements—information, motivation, and behavioral skills—and can comprehensively explain the psychosocial mechanisms of behavior change, making it suitable for promoting HIV testing and safer sexual behaviors.

The primary objective of the study is to evaluate the impact of a social media intervention based on the IMB Model on HIV testing rates and the incidence of high-risk sexual behaviors among MSM in resource-limited areas. The secondary objectives are to explore the intervention's effects on the acceptance rate of syphilis testing, HIV-related knowledge levels, condom use self-efficacy, and attitudes towards safer sexual behaviors.

2. Intervention Design

2.1 Pre-intervention Development Research

(1) In-depth qualitative interviews were conducted with the target population. Through one-on-one, semi-structured individual interviews, we explored MSM users' specific preferences regarding the content format, language style, and presentation medium (e.g., text, images, videos) of HIV testing promotion messages and comprehensive HIV prevention information received via social media such as WeChat. The focus was on identifying what kind of information could effectively motivate their testing intention and safer sexual behaviors.

(2) Based on the results of the qualitative analysis, the research team collaborated closely with core staff from a community-based HIV prevention organization with long-term experience serving MSM populations. Together, they integrated the user preferences identified in the interviews with the three dimensions of the IMB Model to collaboratively create a preliminary prevention message library. Each message in this library was designed to specifically address "Information" (e.g., necessity, methods), "Motivation" (e.g., personal benefits, risk awareness), and "Behavioral Skills" (e.g., practical advice) related to regular HIV testing and consistent condom use.

(3) To further ensure the comprehensibility, appeal, and relevance of the messages, we

subsequently conducted a round of cognitive interviews. We recruited ten MSM who met the study's population characteristics from community venues and asked them to review all materials in the preliminary message library one by one. Participants were requested to comment on and critique the clarity (ease of understanding), level of interest (attractiveness), and personal/group relevance (feeling of closeness to themselves) of each message. The research team systematically collected and analyzed this direct feedback from the target users and accordingly made crucial revisions and improvements to the preliminary message library, such as adjusting obscure terminology, optimizing the narrative style of examples, and enhancing the expressiveness of visual elements.

2.2 Intervention Development

(1) Using the IMB Model as the core framework, the intervention content was designed to cover:

① Information Module: HIV/AIDS-related knowledge, information on Pre-exposure/Post-exposure Prophylaxis (PrEP/PEP).

② Motivation Module: Real-life HIV risk cases, prevention narratives.

③ Behavioral Skills Module: Guidance on safer sexual behaviors, correct condom use, instructions for self-testing kits.

④ Behavior Facilitation Module: Provision of free HIV self-testing kits.

(2) Developed the WeChat mini-program "Rainbow Home" as the intervention platform.

(3) Produced related videos without cultural customization, using Chinese language and Mandarin.

(4) Invited experts in AIDS prevention and control, public health, psychology, and other relevant fields to conduct multiple rounds of review and revision of the intervention content.

2.3 Pilot Study

2.3.1 Participant Recruitment

(1) Recruitment Site: Guangxi Green City Rainbow Activity Center.

(2) Inclusion Criteria:

① Biological sex male; ② Age 18-45 years; ③ Permanent resident of Nanning or planning to reside there for most of the coming year; ④ Had male-to-male sexual behavior in the past 6 months; ⑤ Proficient in using WeChat and willing to bind a study account; ⑥ Tested HIV-negative within the past 3 months (with photographic proof of result); ⑦ Able to understand the study procedures and complete surveys.

(4) Exclusion Criteria: ① Presence of severe psychological, psychiatric, or intellectual disabilities preventing participation; ② Concurrent participation in other intervention studies.

2.3.2 Sample size estimation

Given that this was an exploratory pilot study without a predefined hypothesis, we did not conduct a formal sample- size or power calculation. We will use a one sided

confidence interval approach to estimate the retention rate1. Specifically, this 3-month pilot study is designed with the primary objective of assessing feasibility based on participant retention at the end of the intervention period. We anticipate a retention rate of 90%, with a margin of error of 5%. In other words, a retention rate as low as 85% would be considered acceptable for deeming the pilot feasible and proceeding to a full-scale trial. As the outcome is binary (retained vs not retained), a 95% one-sided confidence interval will be applied to justify the sample size of the pilot study. Accordingly, a minimum of 36 participants was determined to be required for this pilot study. Based on this calculation, we determined that at least 36 participants should be recruited for this study.

$$E = \rho \pm Z\sqrt{\frac{\rho(1-\rho)}{n}}$$

In the above formula, E represents the margin of error, p represents the estimated proportion, n denotes the sample size, and Z is the standard normal deviate associated with the selected confidence interval.

Considering that the primary aim of this pilot study was to test the feasibility and acceptability of the social media intervention, the pilot follow-up period was limited to three months (the intervention period). The 6-month follow-up planned for the full-scale RCT will be carried out after refining the procedures based on this pilot phase.

2.3.3 Intervention Procedure

(1) Grouping: Eligible participants were randomly assigned in a 1:1 ratio to the intervention group or the control group.

(2) Control Group: Received standard HIV/AIDS prevention services (offline outreach, regular testing, monthly peer education).

(3) Intervention Group: In addition to standard services, received video-based task assignments pushed via the "Rainbow Home" mini-program.

(4) Frequency: two to three video tasks were pushed every Sunday at 19:30.

(5) Completion Deadline: Participants must complete learning for each pushed task within one week.

(6) Reminder Mechanism: WeChat push notifications for task reminders.

(7) Intervention time: one month.

(8) Core Content: Videos and interactive materials based on the four modules of the IMB Model.

(9) Follow-up: Baseline and post-intervention follow-ups were conducted in person at the Guangxi Green City Rainbow Activity Center.

2.3.4 Quality Control

① Conducted unified training for community peer educators to ensure standardized recruitment, baseline assessment, and follow-up procedures.

② Monitored participant task completion progress through backend data from the mini-program.

③ Exported and assessed backend monitoring data every two weeks.
④ Regularly checked the completeness of follow-up data.
⑤ Appointed an independent researcher to supervise the implementation of randomization and blinding.

2.3.5 Outcomes

(1) Primary Outcome Measures
① HIV testing rate and HIV self-testing rate.
② Incidence of high-risk sexual behaviors: Defined as not consistently and correctly using condoms or experiencing condom breakage during all sexual acts in the past 3 months.
(2) Secondary Outcome Measures
① Syphilis testing acceptance rate.
② HIV and PrEP/PEP knowledge level (assessed using validated scales).
③ Condom use self-efficacy (assessed using a validated scale).
④ Attitudes towards safer sexual behaviors (assessed using a validated scale).

2.3.6 Statistical Methods

Chi-square tests or Fisher's exact tests were used for comparisons of categorical variables. T-tests or Paired t-tests of variance were used for continuous variables. All analyses were performed using R Studio 4.2.1 or SPSS 26.0, with α set at 0.05.

2.3.7 Ethical Considerations

The study protocol was approved by the Medical Ethics Committee of Nanning Center for Disease Control and Prevention (No.2023014). Written informed consent was provided by all participants. All analytical data were processed anonymously to ensure participant confidentiality. All research staff committed to not disclosing any participant information.

2.3.8 Results

Between March and April 2023, 41 MSM were screened for eligibility after responding to recruitment invitations. Of these, Five participants were excluded from participation: two tested HIV positive, two were unable to commit to the 3-month intervention period, and one due to unfamiliarity with smartphone use. The remaining 36 eligible participants were enrolled in the pilot RCT and completed the baseline assessment procedures. The procedures for participant recruitment, randomization, and follow-up in this pilot RCT are presented in Figure S1.

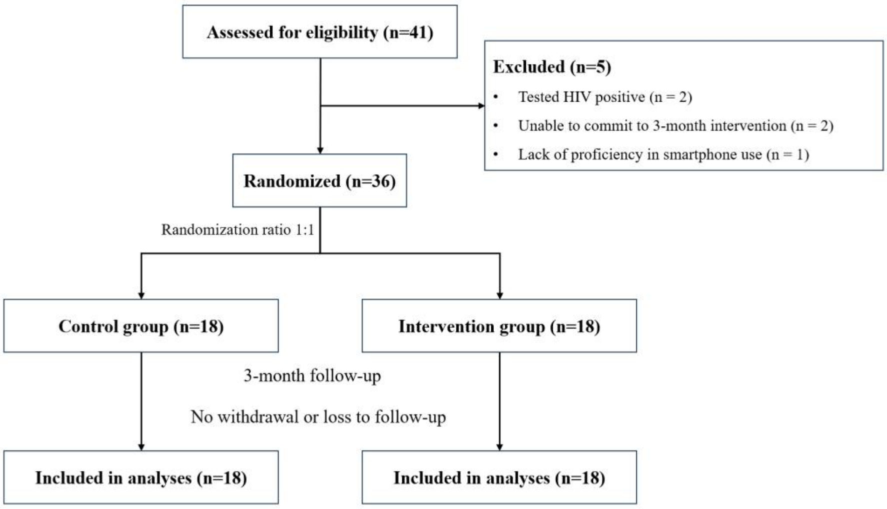

Figure S1. Flow diagram of participant enrollment, randomization, and follow-up in the pilot randomized controlled trial.

During the study period, no participant was lost to follow-up and no new HIV infections were detected among participants. The mean age of participants was 29.0 ± 7.5 years. After randomization, there were no significant differences in demographic or sexual behavior characteristics between the intervention and control groups. Detailed demographic and sexual behavior information is presented in Table S1 and Table S2.

Table S1. Sociodemographic and behavioral,characteristics of study participants

Characteristic	Total [n (%)]	Control group [n (%)]	Intervention group [n (%)]	χ^2	*P*
Age				0.493	0.782
≤ 24	9 (25)	4 (22.2)	5 (27.8)		
25 - 34	22 (61.1)	12 (66.7)	10 (55.6)		
≥ 35	5 (13.9)	2 (11.1)	3 (16.7)		
Household registration				-	1.000[a]
Nanning City	35 (97.2)	18 (100.0)	17 (94.4)		
Other cities within the province	1 (2.8)	0 (0.0)	1 (5.6)		
Ethnicity				0.133	0.716
Han	20 (55.6)	9 (50.0)	11 (61.1)		
Zhuang	16 (44.4)	9 (50.0)	7 (38.9)		
Education level				3.676	0.133[a]
Junior high school or below	2 (5.6)	2 (11.1)	0 (0.0)		
Vocational school / High school	8 (22.2)	2 (11.1)	6 (33.3)		
College / Bachelor's degree or higher	26 (72.2)	14 (77.8)	12 (66.7)		
Occupation				4.406	0.354[a]
Student	2 (5.6)	0 (0.0)	2 (11.1)		
Public institution / Staff	15 (41.7)	8 (44.4)	7 (38.9)		
Farmer / Worker	1 (2.8)	0 (0.0)	1 (5.6)		
Self-employed	13 (36.1)	6 (33.3)	7 (38.9)		

Unemployed	5 (13.9)	4 (22.2)	1 (5.6)		
Monthly income (CNY)				0.657	0.901
< 3000	6 (16.7)	3 (16.7)	3 (16.7)		
3000 - 5000	18 (50.0)	8 (44.4)	10 (55.6)		
> 5000	12 (33.3)	7 (38.9)	5 (27.8)		
Marital status				1.207	1.000[a]
Unmarried	33 (91.7)	16 (88.9)	17 (94.4)		
Married	1 (2.8)	1 (5.6)	0 (0.0)		
Divorced	2 (5.6)	1 (5.6)	1 (5.6)		
Living arrangement				0.280	1.000[a]
Living alone	19 (52.8)	9 (50.0)	10 (55.6)		
Living with family/partner	9 (25)	5 (27.8)	4 (22.2)		
Dormitory / Shared rental	8 (22.2)	4 (22.2)	4 (22.2)		
Sexual orientation				1.394	0.711[a]
Homosexual	25 (69.4)	12 (66.7)	13 (72.2)		
Bisexual	10 (27.8)	6 (33.3)	4 (22.2)		
Uncertain	1 (2.8)	0 (0.0)	1 (5.6)		
Sexual role				3.635	0.194[a]
Insertive role	9 (25)	7 (38.9)	2 (11.1)		
Receptive role	13 (36.1)	5 (27.8)	8 (44.4)		
Versatile	14 (38.9)	6 (33.3)	8 (44.4)		
Age at first anal sex (years)				0.111	0.738
≤ 18	17 (47.2)	8 (44.4)	9 (50.0)		
> 18	19 (52.8)	10 (55.6)	9 (50.0)		
Number of regular partners				0.131	0.717
0	11 (30.6)	6 (33.3)	5 (27.8)		
1 - 2	25 (69.4)	12 (66.7)	13 (72.2)		
Number of casual partners				0.131	0.717
0	11 (30.6)	6 (33.3)	5 (27.8)		
1 - 2	25 (69.4)	12 (66.7)	13 (72.2)		
Awareness of partner's HIV status				2.786	0.095
No	19 (52.8)	7 (38.9)	12 (66.7)		
Yes	17 (47.2)	11 (61.1)	6 (33.3)		
Sexting				0.120	0.729
No	13 (36.1)	7 (38.9)	6 (33.3)		
Yes	23 (63.9)	11 (61.1)	12 (66.7)		
Popper/alkyl nitrite inhalant use				0.000	1.000
No	22 (61.1)	11 (61.1)	11 (61.1)		
Yes	14 (38.9)	7 (38.9)	7 (38.9)		
Consistent condom use				3.010	0.083
Yes	23 (63.9)	14 (77.8)	9 (50.0)		
No	13 (36.1)	4 (22.2)	9 (50.0)		
HIV self-testing				0.444	0.505
No	18 (50.0)	8 (44.4)	10 (55.6)		

Yes	18 (50.0)	10 (55.6)	8 (44.4)		
Syphilis test result				0.394	0.530
Negative	31 (86.1)	16 (88.9)	15 (83.3)		
Positive	5 (13.9)	2 (11.1)	3 (16.7)		
HIV testing				-	-
No	0 (0.0)	0 (0.0)	0 (0.0)		
Yes	36 (100.0)	18 (100.0)	18 (100.0)		

Note: a represents Fisher's exact test.

Table S2. Baseline comparison of knowledge and behavior scores between the intervention and control groups

	Total (mean ± SD)	Control group (mean ± SD)	Intervention group (mean ± SD)	statistic	*P*
Sexual behavior attitude score	43.2 ± 8.0	43.1 ± 7.9	43.4 ± 8.3	-0.144	0.086
Condom use self efficacy score	27.9 ± 5.2	27.1 ± 4.6	28.7 ± 5.8	-0.928	0.360
AIDS knowledge score	12.6 ± 3.2	13.5 ± 2.8	11.7 ± 3.4	1.769	0.086
PrEP knowledge score	4.6 ± 2.1	5.1 ± 1.8	4.1 ± 2.3	1.373	0.179
PEP knowledge score	5.6 ± 1.7	5.4 ± 1.5	5.8 ± 1.9	-0.790	0.435

After the 3-month new media based intervention, an immediate follow-up survey was conducted among participants in both groups. Compared with the control group, participants in the intervention group showed a lower frequency of stimulant use during sexual activity and a higher rate of HIV self-testing (Table S3). Regarding the behavioral and knowledge assessment scales, the intervention group demonstrated a better level of knowledge of PrEP (P = 0.037, Figure S2), while the mean score for AIDS-related knowledge was higher but not statistically significant ($P = 0.690$). No significant between-group differences were observed for other outcome measures.

Table S3. Comparison of sexual behavior characteristics and HIV testing status between the intervention group and the control group among study participants after 3-month follow-up

Characteristic	Group		χ^2	*P*
	Control group (n=18)	Intervention group (n=18)		
Number of regular partners			0.554	0.711
0	6.0 (33.3)	4.0 (22.2)		
1-2	12.0 (66.7)	14.0 (77.8)		
Number of casual partners			2.107	0.367
0	7.0 (38.9)	10.0 (55.6)		
1-2	9.0 (50.0)	6.0 (33.3)		
≥3	2.0 (11.1)	2.0 (11.1)		
Consistent condom use			1.084	0.489
No	8.0 (44.4)	5.0 (44.4)		
Yes	10.0 (55.6)	13.0 (55.6)		
Sexting			0.131	1.000
No	6.0 (33.3)	5.0 (27.8)		
Yes	12.0 (66.7)	13.0 (72.2)		

Popper/alkyl nitrite inhalant use			5.461	**0.044**
No	6.0 (33.3)	13.0 (72.2)		
Yes	12.0 (66.7)	5.0 (27.8)		
HIV testing			1.172	0.603[a]
No	1.0 (5.6)	3.0 (16.7)		
Yes	17.0 (94.4)	15.0 (83.3)		
HIV self-testing			7.111	**0.018**
No	13.0 (72.2)	5.0 (27.8)		
Yes	5.0 (27.8)	13.0 (72.2)		

Note: a represents Fisher's exact test.

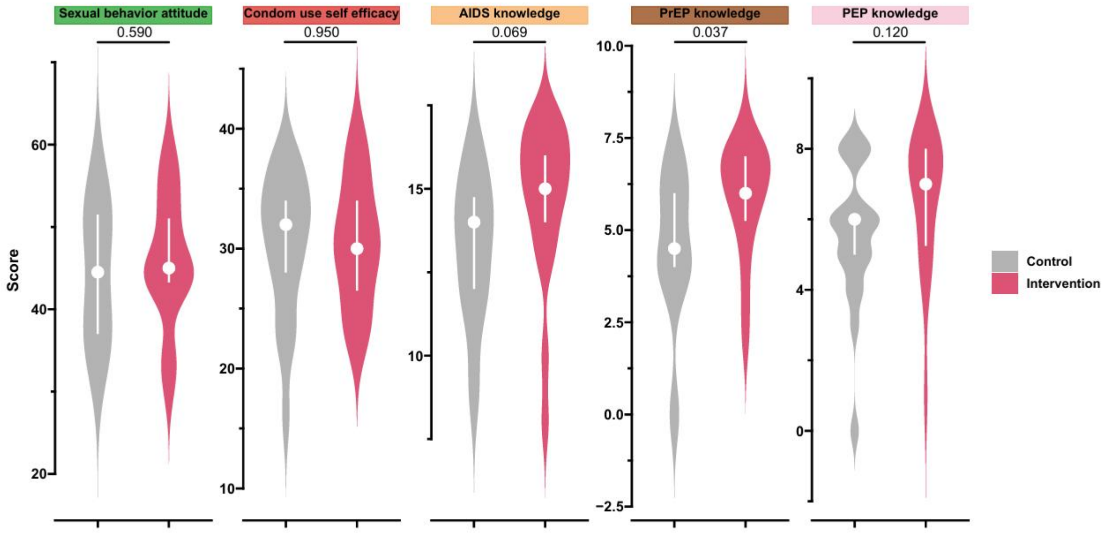

Figure S2. Comparison of post-intervention behavioral and knowledge scores between the control and intervention groups.

Within the intervention group, paired analyses were conducted to compare scale scores before and after the 3-month new media based intervention. Significant increases were observed in AIDS knowledge and PrEP knowledge scores after the intervention, whereas no significant changes were observed in other outcome measures.

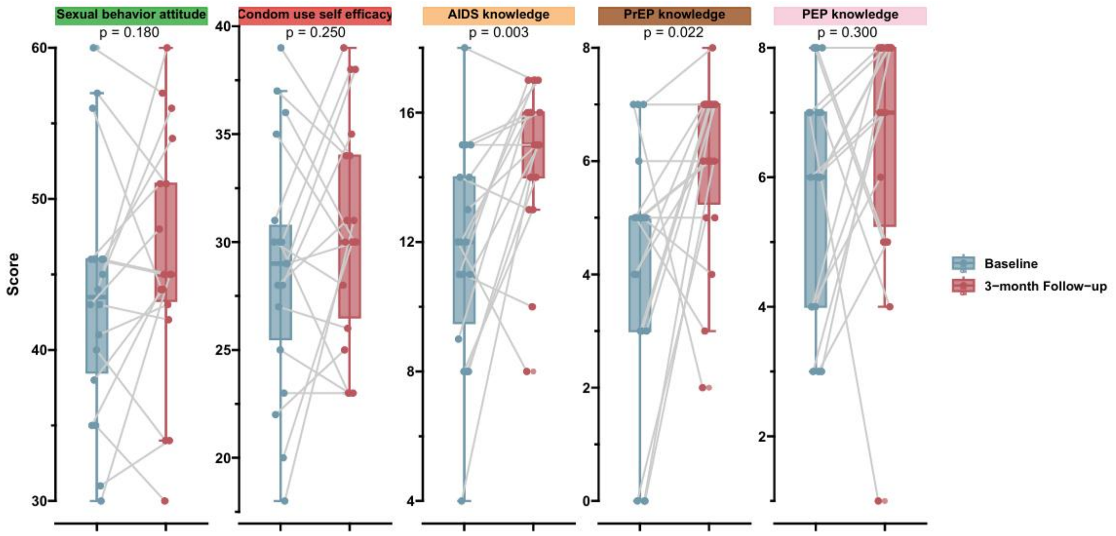

Figure S3. Changes in behavioral and knowledge scores within the intervention group before and after the 3-month new media based intervention

After the intervention, qualitative interviews were conducted with participants in the intervention group to obtain feedback on their experiences using the WeChat mini-program. Most participants noted that the intervention was comprehensive, covering multiple aspects of HIV prevention and sexual health. They appreciated the accessible educational materials, timely reminders, and interactive features that supported self-learning and reflection on safer-sex practices. Overall, the new-media based intervention enhanced their awareness of HIV self-testing, PrEP, and PEP, motivating them to adopt or consider preventive behaviors. The complete list of qualitative comments can be found in Table S4.

**Table S4.** Selected feedback from participants in the intervention group (n=10)
◆ *Before the intervention, I barely knew about PrEP. Now I understand how it works and where to get it.*
◆ *The videos were engaging and made it easier to discuss sexual health topics without embarrassment.*
◆ *I would prefer more practical information, such as where to obtain free condoms or PrEP.*
◆ *Regular messages on social media reminded me to pay attention to my health and get tested.*
◆ *The information about HIV self-testing was very useful; I tried it for the first time after joining the project.*
◆ *The WeChat mini-program made complex HIV-related knowledge easier to understand through concise language and visual presentations.*
◆ *Nothing. It was very comprehensive.*
◆ *When I actually saw the medical treatment process of AIDS patients, I felt that I would go back on high-risk sexual behavior before, and I admit that it worked.*
◆ *It turns out that the AIDS virus is not as terrible as I imagined. I naively thought that I could not have children again.*
◆ *I used to take stimulants quite often, but through the program I learned about their health risks and decided to reduce my use.*
◆ *As an MSM who has often felt marginalized, I felt happy that I could share my story with the staff through the online platform.*

2.3.9 Discussion

In this 3-month pilot RCT, a new-media based intervention developed under the IMB model demonstrated good feasibility and preliminary effectiveness. Between March and April 2023, MSM were recruited through peer educators. The recruitment was completed in less than one month from initiation to completion, and notably, no participant was lost to follow-up during the entire three-month intervention period, indicating a higher-than-expected level of adherence. Immediately after the intervention, all participants completed a follow-up assessment. Compared with the control group, participants in the intervention arm reported a significant reduction in the frequency of stimulant use during sexual activity, as well as a downward but not statistically significant-trend in the frequency of recent high-risk sexual behavior. Moreover, HIV self-testing uptake increased significantly in the intervention group, accompanied by a marked improvement in HIV-related knowledge. These findings are consistent with results from previous WeChat-based interventions conducted in Anhui and Guangdong provinces, which also reported higher HIV testing rates among participants exposed to social media interventions. No significant changes were observed in attitudes toward safer sex between groups, suggesting that such attitudes among MSM may remain relatively stable and deeply influenced by long-standing sociocultural and personal factors, thus requiring longer or more intensive interventions to effect meaningful changes. Future studies with extended intervention and follow-up durations are needed to further evaluate the long-term impact on sexual behavior attitudes.

Persistent social stigma and discrimination, particularly in resource-limited settings, often limit access to or reduce the effectiveness of traditional sexual health education and counseling for MSM. In contrast, new-media-based interventions overcome geographical and social barriers by providing private, flexible, and accessible channels through which individuals can obtain sexual health information at any time. By using engaging formats such as graphics, animations, and short videos, these interventions capture attention, promote interactivity, and enhance comprehension and retention of health messages. Our intervention was developed within the framework of the IMB model, which posits that health behavior change is jointly determined by adequate information, personal motivation, and behavioral skills. The observed improvements in HIV self-testing uptake and HIV-related knowledge among participants in the intervention group support the mechanism proposed by this model, demonstrating how information delivery and motivational enhancement through new media can translate into measurable behavioral outcomes. Taken together, these findings highlight both the theoretical soundness and practical feasibility of IMB-based social media interventions, suggesting considerable potential for scaling up such strategies to reduce high-risk sexual behaviors and promote HIV prevention among MSM populations.

This pilot randomized controlled trial has several limitations that should be acknowledged. First, our findings were based on one-to-one interviews conducted by

research staff, which might have introduced interviewer-related variability, particularly in the collection of sensitive information such as sexual behaviors. During follow-up, social desirability bias may have influenced participants' responses, potentially leading to under-reporting of high-risk behaviors and consequent reporting bias. Second, given the unique characteristics of the MSM population, the use of respondent-driven sampling might have resulted in social connections between participants in the intervention and control groups, leading to possible contamination across study arms. To minimize such cross-group contamination in future full-scale RCT, we plan to implement stricter management procedures for intervention access. Specifically, the *Rainbow Home* app will require a unique entry password accessible only through research staff, and the password will be refreshed periodically after each authorized access to ensure group integrity and intervention fidelity.

2.3.10 Conclusion

This study demonstrated that a full-scale RCT of a new media based intervention among MSM is feasible. The intervention may have a positive effect on reducing high-risk sexual behaviors and improving HIV self-testing uptake.

2.3.11 Caution

Due to the pilot nature of this study, these results must be interpreted with caution until replicated in a full-scale study.

2.3.12 User Feedback Collection

Upon completion of the intervention, we conducted exit interviews with participants to systematically gather their feedback on the usage experience of the WeChat mini-program and suggestions for improvement. The interview content primarily included the following aspects:

① Overall User Experience: Participants were asked to describe their overall feelings about using the WeChat mini-program.

② Evaluation of Information Content: Participants were invited to provide specific comments and critiques on the various types of information received (e.g., text, videos, etc.).

③ Collection of Improvement Suggestions: Participants were encouraged to offer general recommendations for enhancing user engagement and retention in the future.

3. Refinement of Intervention Content and Procedures

Based on the aforementioned results and user feedback, the intervention content and procedures were refined, with a primary focus on detailed improvements.
