# Supplementary material for: Social Media Intervention Based on the Information-Motivation-Behavioral Skills Model Promotes HIV Testing and Reduces High-Risk Behaviors Among Men Who Have Sex With Men in Resource-Limited Settings in China: Randomized Controlled Trial
Source: J Med Internet Res. 2026 Apr 7;28:e84279. doi: 10.2196/84279 (PMC13055935; doi:10.2196/84279)

Operational process of WeChat mini-program

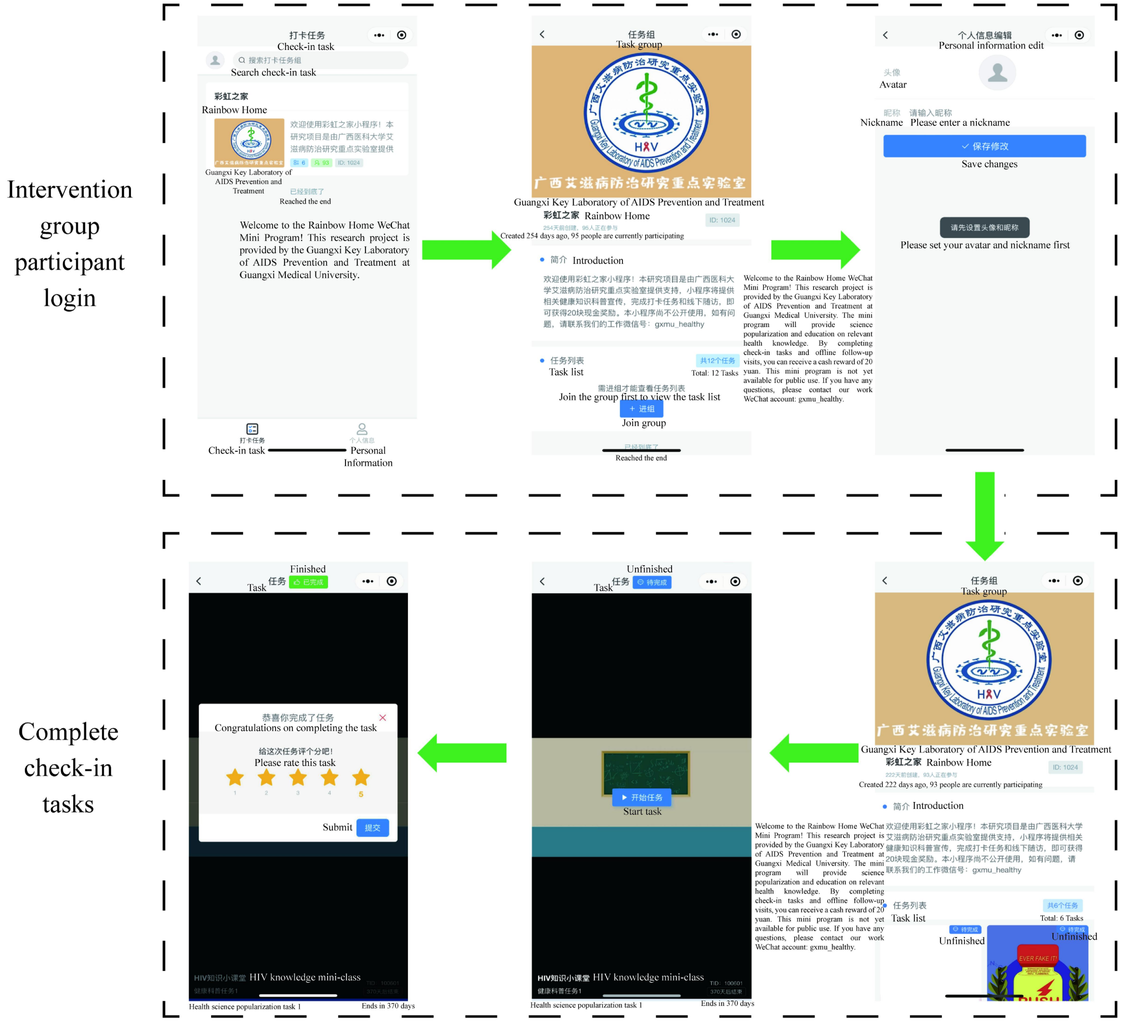

Supplement: Multimedia Appendix 2 [file jmir-v28-e84279-s002.pdf]
