# Supplementary material for: Social Media Intervention Based on the Information-Motivation-Behavioral Skills Model Promotes HIV Testing and Reduces High-Risk Behaviors Among Men Who Have Sex With Men in Resource-Limited Settings in China: Randomized Controlled Trial
Source: J Med Internet Res. 2026 Apr 7;28:e84279. doi: 10.2196/84279 (PMC13055935; doi:10.2196/84279)
Supplement: Multimedia Appendix 3 [file jmir-v28-e84279-s003.pdf]

Video tasks of the intervention plan based on the information-motivation-behavioral skills model

No	Video title	Duration (min)	Main content	Module
1	Introduction to sexually transmitted infections (HIV) and how they are transmitted	5	This session provides an overview of HIV and other sexually transmitted infections (STIs), including syphilis, gonorrhea, human papillomavirus (HPV), and monkeypox, summarizing their main characteristics, transmission routes, symptoms, potential health outcomes, and the differences in pathogens, clinical manifestations, and overall health impacts. Participants will learn to distinguish HIV from other STIs by recognizing their specific symptoms, diagnostic methods, and prevention strategies. The session also emphasizes the importance of condom use and regular STI screening as measures for preventing infection.	Information
2	Preventing HIV infection and immediate actions after risk exposure	6	This section introduces effective strategies to prevent HIV infection, such as consistent condom use, regular HIV testing, and outlines immediate actions to take after potential exposure to the virus.	Information and behavioral skill
3	The strategy for HIV prevention: pre-exposure prophylaxis (PrEP) and post-exposure prophylaxis (PEP).	8	This session introduces the concepts of PrEP and PEP, explaining how they reduce the risk of HIV infection and outlining the important differences between the two approaches. Participants will learn who is eligible to use PrEP or PEP, when each should be initiated, and the recommended duration of medication.	Information
4	The hidden risks of sexual enhancers	5	This course introduces the risks associated with the use of sexual enhancers, particularly inhalants such as poppers (Rush). Although these substances may temporarily increase excitement or reduce anxiety, their misuse can lead to adverse effects such as fluctuations in blood pressure, dizziness, and even cardiovascular complications. More importantly, sexual enhancers often lower inhibitions and	Information

			impair judgment, which can result in unprotected sex, multiple sexual partners, and an increased risk of HIV or other sexually transmitted infections. The session aims to raise awareness of the physical and psychological harms of these substances and encourage safer sexual behaviors.	
5	Access and use HIV self-testing kits through online platforms	5	This section introduces how participants can obtain and use HIV self-testing kits conveniently through online platforms. Participants will learn how to identify reliable sources such as government health websites, community organizations, or certified online pharmacies that provide free or low-cost self-testing kits delivered privately by mail. The video explains the steps for proper use of these kits, including verifying the package's integrity, following the instructions for sample collection, reading and interpreting the results accurately, and understanding what to do next. If the test result is positive, viewers are advised to contact their local Center for Disease Control (CDC) for confirmatory testing or reach out to peer educators affiliated with our organization for further guidance and support. If the result is negative, they are encouraged to continue regular testing and maintain safe sexual practices.	Motivation and behavioral skill
6	Real- Life cases sharing: experiences of patient with HIV	8	This video features the real- life case of an anonymous individual living with HIV, sharing his experience from before infection to diagnosis and beyond. The case highlights different stages—from the initial emotional reactions following diagnosis to the growing understanding of the importance of early testing, adherence to regular treatment, and the value of social support. The case- sharing segment aims to foster understanding, empathy, and awareness, encouraging	Motivation

			viewers to support those affected and to adopt safer health behaviors themselves.	
7	How to talk about safer sex with your partner	6	This section focuses on how to communicate effectively with sexual partners and negotiate safer sexual behaviors. Through examples and role-play scenarios, participants will learn how to initiate these conversations, for example, by expressing personal boundaries, suggesting condom or lubricant use in a positive way, and handling situations where a partner may resist or avoid such discussions. The video emphasizes that good communication is not only about protection, but also about building trust, mutual respect, and decision-making equality in relationships. Before intimacy, both partners should talk honestly about their HIV status, recent testing history, and preferences for preventive measures. It is especially important to reach a clear agreement on consistent condom use throughout the entire sexual encounter to reduce the risk of HIV and other sexually transmitted infections.	Motivation and behavioral skill
8	How to cope with HIV stigma and discrimination	5	Stigma and discrimination against men who have sex with men (MSM) often arise from fear or misinformation about HIV transmission, as well as limitations rooted in traditional cultural attitudes and social norms. This video aims to help participants understand what stigma is, how it develops, and how to respond to it effectively. The video emphasizes the right of every individual to be treated with dignity and respect. In coping with stigma, MSM are advised to seek emotional and social support from trusted peers, community organizations, and professional counselors. By improving self-acceptance and confidence, individuals can face stigma and	Motivation and behavioral skill

			discrimination with positivity.	
9	Mental health and emotional well-being	5	Many men who have sex with men (MSM) experience internal conflicts or self-doubt due to long-term social pressure and stigma. Such feelings can easily develop into anxiety, depression, or a sense of loneliness, which negatively affect mental health and daily life. This session encourages participants to build self-acceptance and positive self-identity, to talk openly with trusted friends or counselors, and to engage in activities that bring relaxation and confidence. Regular self-care, such as sufficient rest, balanced nutrition, and physical exercise, can also improve emotional stability.	Motivation and behavioral skill
10	Strengthening self-acceptance, and self-confidence of Identity	5	Every person deserves respect, dignity, and love, regardless of sexual orientation or HIV status. Many MSM grow up hearing negative messages about their sexual orientation or gender expression. Such internalized stigma can quietly affect emotional well-being, relationships, and even decisions about sexual safety. This video helps participants learn practical strategies to strengthen self-acceptance and confidence. They are encouraged to focus on their strengths, acknowledge their emotions without judgment, and connect with supportive people or communities.	Motivation
11	Daily life and self-care for patient with HIV	6	This video introduces how a patient who has lived with HIV for many years maintains a fulfilling and balanced life. He goes to work, builds relationships, exercises, travels, and enjoys ordinary routines just like everyone around him. By taking medication regularly, keeping medical appointments, and caring for his emotional well-being, he demonstrates that HIV does not define his identity or limit his future. Participants will learn the importance of taking	Information and Motivation

			antiretroviral therapy (ART) on time every day to keep the viral load low and protect the immune system.	
12	Coping with reproductive Issues while patient with HIV	8	Modern medical advances have made it possible for people living with HIV to build families and have children just like anyone else. This video explains how individuals and couples can plan for parenthood while preventing HIV transmission to their partners and future babies. For HIV-positive men, maintaining an undetectable viral load through consistent antiretroviral therapy (ART) greatly reduces the risk of transmission to an HIV-negative partner. It also introduces options such as preconception counseling, viral load monitoring, and other clinical methods recommended by healthcare professionals.	Information and behavioral skill
