# Supplementary material for: Social Media Intervention Based on the Information-Motivation-Behavioral Skills Model Promotes HIV Testing and Reduces High-Risk Behaviors Among Men Who Have Sex With Men in Resource-Limited Settings in China: Randomized Controlled Trial
Source: J Med Internet Res. 2026 Apr 7;28:e84279. doi: 10.2196/84279 (PMC13055935; doi:10.2196/84279)
Supplement: Multimedia Appendix 4 [file jmir-v28-e84279-s004.pdf]

Definitions of outcomes and methods of outcome assessment

1 HIV and Syphilis Testing and Related Service Utilization

Included: HIV and Syphilis testing in the past 3 months; history of HIVST; willingness to use self-test kits; PrEP use history and willingness to use; PEP use history; utilization of HIV counseling/intervention services.

2 Sexual Behavior Characteristics

Included: engagement in "sexting" (defined as sex-seeking behavior and/or sexually explicit communication via online social or entertainment platforms, which may include suggestive or explicit text, audio, images, or videos) [1] in the past 3 months; sexual orientation; sexual role preference; type(s) of sexual partners; number of sexual partners in the past 3 months; awareness of partners' HIV status; occurrence of high-risk sexual behavior (defined as engaging in anal intercourse with a male partner without consistent condom use throughout the act or experiencing condom breakage during intercourse). Also included frequency of recreational drug and erectile dysfunction drug use in the past 3 months.

3 HIV-Related Knowledge

Assessed using the internationally validated HIV-Knowledge Questionnaire-18 (HIV-KQ-18) (Cronbach's $\alpha = 0.75\text{-}0.89$) [2]. This 18-item scale includes 10 items on sexual transmission and 8 on non-sexual transmission, all are true/false questions. Each item is scored as 1 for correct and 0 for incorrect or "Don't know". The total score ranges from 0 to 18, with higher scores indicating better HIV-related knowledge. The questionnaire used for the assessment is as follows:

	Correct	Incorrect	Don't know
1. Coughing or sneezing does not transmit hiv	①	②	③
2. Sharing a cup to drink water with an hiv carrier can lead to hiv infection	①	②	③
3. Withdrawing the penis before ejaculation can prevent female partners from getting infected during intercourse	①	②	③
4. Women engaging in anal sex with men may contract hiv	①	②	③
5. Showering or cleaning genitals/private parts after intercourse prevents hiv infection	①	②	③
6. All babies born to pregnant women with hiv are born with aids	①	②	③
7. People infected with hiv quickly show severe signs of infection	①	②	③
8. there is currently a vaccine that prevents hiv infection in adults	①	②	③
9. ritualistic kissing with an hiv-infected partner may lead to aids infection	①	②	③
10. women cannot contract hiv during menstrual sex	①	②	③
11. there is currently a female condom that helps reduce women's risk of hiv infection	①	②	③
12. natural skin condoms are more effective than latex condoms in preventing hiv	①	②	③
13. taking routine antibiotics prevents hiv infection	①	②	③
14. having multiple sexual partners increases the risk of hiv infection	①	②	③
15. hiv infection can be detected one week after sexual intercourse	①	②	③
16. sharing a hot tub or swimming with an hiv carrier may lead to hiv infection	①	②	③
17. oral sex can transmit hiv	①	②	③
18. using condoms with vaseline or baby oil reduces the risk of hiv infection	①	②	③

4 PrEP and PEP Knowledge

Assessed using questionnaires based on Shi Anxia's survey in China [3], comprising 8 true/false items each for PrEP and PEP knowledge. Scoring was identical to 2.5.4 (1 point per correct answer). Total scores for PrEP and PEP knowledge each ranged from 0 to 8, with higher scores indicating better knowledge. In this study, both PrEP and PEP knowledge scales demonstrated good reliability (Cronbach's $\alpha = 0.765$ and 0.764, respectively) and validity (KMO = 0.808 and 0.797, respectively). The questionnaire used for the assessment is as follows:

PrEP Knowledge

	Correct	Incorrect	Don't know
1. Suitable for men who have sex with men who are confirmed to be not infected with hiv and are at risk of infection	①	②	③
2. Suitable for men who have sex with men who cannot consistently use condoms during sexual activity	①	②	③
3. Side effects such as dizziness, headaches, nausea, vomiting, and loss of appetite may occur after using prep medication	①	②	③
4. The general dosing method for PrEP medication is once daily	①	②	③
5. Can serve as an alternative method for those who never use condoms	①	②	③
6. PrEP provides some preventive effect against other sexually transmitted infections	①	②	③
7. Using prep can completely block hiv infection	①	②	③
8. Individuals using prep must undergo regular follow-up testing	①	②	③

PEP Knowledge

	Correct	Incorrect	Don't know
1. Suitable for men who have sex with men who are confirmed to be not infected with HIV and are at risk of infection	①	②	③
2. Suitable for men who have sex with men who cannot consistently use condoms during sexual activity	①	②	③
3. Side effects such as dizziness, headaches, nausea, vomiting, and loss of appetite may occur after using PEP medication	①	②	③
4. The first dose of PEP should be taken within 72 hours after exposure, and the medication should be continued for 28 days	①	②	③
5. Using PEP can completely block HIV infection	①	②	③
6. PEP provides some preventive effect against other sexually transmitted infections	①	②	③
7. Regular follow-up testing is required after using PEP medication	①	②	③
8. Continuous pre-exposure prophylaxis (PrEP) provides greater protection than repeated post-exposure prophylaxis (PEP)	①	②	③

5 Attitudes Towards Safer Sex

Assessed using a questionnaire based on the survey used by Yifei Hu et al. in China

(Cronbach's α = 0.95) [4]. This 15-item scale uses a 4-point scale ("Strongly Disagree", "Disagree", "Agree", "Strongly Agree"), scored 1-4, with some reverse-scored items. The total score ranges from 15 to 60, with higher scores indicating more positive attitudes towards safer sex. Based on the scale's scoring criteria and using the 75th percentile cut-off, a total score >53 was defined as a high level of safer sex attitudes in this study. The questionnaire used for the assessment is as follows:

*Reverse scored items

	Strongly Disagree	Disagree	Agree	Strongly Agree
1 Most gay men I meet only engage in safer sex	①	②	③	④
*2 I have difficulty letting my sexual partners know that I want safer sex	①	②	③	④
3 I am able to avoid behaviors that might put me at risk of HIV infection	①	②	③	④
4 My friends think using condoms is important	①	②	③	④
5 I can consistently use condoms with regular sexual partners	①	②	③	④
6 I can consistently use condoms with casual sexual partners	①	②	③	④
*7 I find it difficult to maintain safer sex with a man who strongly arouses me sexually	①	②	③	④
*8 I find it difficult to maintain safer sex when climaxing or drunk	①	②	③	④
*9 Now that new combination anti-HIV drugs are available, I am less worried about the risks of condomless anal sex	①	②	③	④
*10 Someone could use their HIV-negative status to persuade me to forgo condom use	①	②	③	④
11 If I engage in high-risk HIV behavior, I am confident I can correct it and quickly return to safer sex practice	①	②	③	④
12 I am confident that I can insist on using condoms even if my partner is reluctant	①	②	③	④
*13 I find it difficult to tell a sexual partner "I won't have anal sex without a condom"	①	②	③	④
14 I am confident that I can use condoms with any sexual partner	①	②	③	④
15 My friends encourage me to practice safer sex	①	②	③	④

6 Condom Use Self-Efficacy

Assessed using a questionnaire based on the online survey used by Cheng Wang et al. in

China (Cronbach's α = 0.82) [5]. This 8-item scale uses a 5-point Likert scale ("Strongly Disagree", "Disagree", "Neutral", "Agree", "Strongly Agree"), scored 1-5. The total score ranges from 8 to 40, with higher scores indicating greater condom use self-efficacy. Based on the scale's scoring criteria and using the 75th percentile cut-off, a total score >35 was defined as a high level of condom use self-efficacy in this study. The questionnaire used for the assessment is as follows:

	Strongly Disagree	Disagree	Neutral	Agree	Strongly Agree
1 If I don't have a condom when dating, I will find a way to buy one	①	②	③	④	⑤
2 I am willing to discuss condom use with my partner before sexual activity	①	②	③	④	⑤
3 I am comfortable letting my primary partner know that I want to use condoms during sex	①	②	③	④	⑤
4 I am comfortable letting my casual partner know that I want to use condoms during sex	①	②	③	④	⑤
5 I am confident that I can refuse to have sex with a partner who doesn't want me to use condoms	①	②	③	④	⑤
6 I am confident that I can use condoms during foreplay or have my partner use them	①	②	③	④	⑤
7 I feel very uncomfortable when I know others have negative opinions about my sexual orientation	①	②	③	④	⑤
8 I am confident that I can use condoms during sex without "killing the mood"	①	②	③	④	⑤

References

[1] Gassó AM, Mueller-Johnson K, Montiel I. Sexting, Online Sexual Victimization, and Psychopathology Correlates by Sex: Depression, Anxiety, and Global Psychopathology. Int J Environ Res Public Health 2020;17:1018. https://doi.org/10.3390/ijerph17031018.

[2] Carey MP, Schroder KEE. Development and psychometric evaluation of the brief HIV Knowledge Questionnaire. AIDS Educ Prev 2002;14:172–82.

https://doi.org/10.1521/aeap.14.2.172.23902.

[3] Shi AX. Barriers and countermeasures to HIV pre-exposure prophylaxis among men who have sex with men [dissertation]. Anhui Medical University; 2020. https://doi.org/10.26921/d.cnki.ganyu.2020.001212

[4] Hu Y, Lu H, Raymond HF, et al. Measures of condom and safer sex social norms and stigma towards HIV/AIDS among Beijing MSM. AIDS Behav 2014;18:1068–74. https://doi.org/10.1007/s10461-013-0609-7.

[5] Wang C, Tucker JD, Liu C, et al. Condom use social norms and self-efficacy with different kinds of male partners among Chinese men who have sex with men: results from an online survey. BMC Public Health 2018;18:1175. https://doi.org/10.1186/s12889-018-6090-5.
